# Supplementary material for: Changing trends in traumatic spinal cord injury in an aging society: Epidemiology of 1152 cases over 15 years from a single center in Japan
Source: PLoS One. 2024 May 16;19(5):e0298836. doi: 10.1371/journal.pone.0298836 (PMC11098516; doi:10.1371/journal.pone.0298836)
Supplement: S1 Table — (DOCX) [file pone.0298836.s002.docx]

**Supplemental Table1. The relationship between the level of injury and alcohol consumption at the time of injury**

|  | Neurological level of injury | | | | |
| --- | --- | --- | --- | --- | --- |
| Characteristics | C1-C4 | C5-C8 | T1-T6 | T7-T12 | L1-L5 |
| Alcohol consumption at injury |  | | | | |
| Yes (n, %) | 122 (65.9) | 48 (25.9) | 0  (0) | 5  (2.7) | 10 (5.4) |
| No (n, %) | 394 (41.4) | 254 (26.7) | 36 (3.8) | 125 (13.1) | 143 (15.0) |
