# Supplementary material for: Changing trends in traumatic spinal cord injury in an aging society: Epidemiology of 1152 cases over 15 years from a single center in Japan
Source: PLoS One. 2024 May 16;19(5):e0298836. doi: 10.1371/journal.pone.0298836 (PMC11098516; doi:10.1371/journal.pone.0298836)
Supplement: S2 Table — (DOCX) [file pone.0298836.s003.docx]

**Supplemental table 2. Weekday variations in injuries and the number of TSCI cases per month based on the date of injury**

|  | Monday | Tuesday | Wednesday | Thursday | Friday | Saturday | Sunday |
| --- | --- | --- | --- | --- | --- | --- | --- |
| 2005-2009 | 40 | 47 | 47 | 44 | 51 | 50 | 55 |
| 2010-2013 | 33 | 44 | 32 | 40 | 32 | 39 | 30 |
| 2014-2017 | 47 | 38 | 47 | 36 | 45 | 45 | 47 |
| 2018-2021 | 34 | 30 | 30 | 46 | 47 | 38 | 38 |
