# Supplementary material for: Changing trends in traumatic spinal cord injury in an aging society: Epidemiology of 1152 cases over 15 years from a single center in Japan
Source: PLoS One. 2024 May 16;19(5):e0298836. doi: 10.1371/journal.pone.0298836 (PMC11098516; doi:10.1371/journal.pone.0298836)
Supplement: S3 Table — (DOCX) [file pone.0298836.s004.docx]

**Supplemental table 3. Weekday variation in injuries and the percentage of TSCI cases per month based on the date of injury**

|  | Monday | Tuesday | Wednesday | Thursday | Friday | Saturday | Sunday |
| --- | --- | --- | --- | --- | --- | --- | --- |
| 2005-2009 | 12 | 14.1 | 14.1 | 13.2 | 15.3 | 15 | 16.5 |
| 2010-2013 | 13.2 | 17.6 | 12.8 | 16 | 12.8 | 15.6 | 12 |
| 2014-2017 | 15.4 | 12.5 | 15.4 | 11.8 | 14.8 | 14.8 | 15.4 |
| 2018-2021 | 12.9 | 11.4 | 11.4 | 17.5 | 17.9 | 14.4 | 14.4 |
| Mean | 13.4 | 13.9 | 13.4 | 14.6 | 15.2 | 14.9 | 14.6 |
| P value | 0.5127 | 0.1843 | 0.5786 | 0.3588 | 0.3647 | 0.815 | 0.6951 |
