# Supplementary material for: Changing trends in traumatic spinal cord injury in an aging society: Epidemiology of 1152 cases over 15 years from a single center in Japan
Source: PLoS One. 2024 May 16;19(5):e0298836. doi: 10.1371/journal.pone.0298836 (PMC11098516; doi:10.1371/journal.pone.0298836)
Supplement: S4 Table — (DOCX) [file pone.0298836.s005.docx]

**Supplemental table 4. Seasonal variations in injuries and the number of TSCI cases per month based on the date of injury**

|  | Jan | Feb | Mar | Apr | May | Jun | Jul | Aug | Sep | Oct | Nov | Dec | Total |
| --- | --- | --- | --- | --- | --- | --- | --- | --- | --- | --- | --- | --- | --- |
| 2005-2009 | 16 | 19 | 26 | 25 | 30 | 36 | 36 | 30 | 25 | 25 | 39 | 27 | 334 |
| 2010-2013 | 26 | 16 | 28 | 23 | 21 | 24 | 19 | 20 | 16 | 21 | 18 | 18 | 250 |
| 2014-2017 | 32 | 21 | 24 | 26 | 20 | 25 | 25 | 20 | 25 | 40 | 19 | 28 | 305 |
| 2018-2021 | 24 | 9 | 17 | 19 | 17 | 19 | 19 | 22 | 31 | 32 | 31 | 23 | 263 |
