# Supplementary material for: Changing trends in traumatic spinal cord injury in an aging society: Epidemiology of 1152 cases over 15 years from a single center in Japan
Source: PLoS One. 2024 May 16;19(5):e0298836. doi: 10.1371/journal.pone.0298836 (PMC11098516; doi:10.1371/journal.pone.0298836)
Supplement: S5 Table — (DOCX) [file pone.0298836.s006.docx]

**Supplemental table 5. Seasonal variations in injuries and the percentage of TSCI cases per month based on the date of injury**

|  | Jan | Feb | Mar | Apr | May | Jun | Jul | Aug | Sep | Oct | Nov | Dec |
| --- | --- | --- | --- | --- | --- | --- | --- | --- | --- | --- | --- | --- |
| 2005-2009 | 4.8 | 5.7 | 7.8 | 7.5 | 9 | 10.8 | 10.8 | 9 | 7.5 | 7.5 | 11.7 | 8.1 |
| 2010-2013 | 10.4 | 6.4 | 11.2 | 9.2 | 8.4 | 9.6 | 7.6 | 8 | 6.4 | 8.4 | 7.2 | 7.2 |
| 2014-2017 | 10.5 | 6.9 | 7.9 | 8.5 | 6.6 | 8.2 | 8.2 | 6.6 | 8.2 | 13.1 | 6.2 | 9.2 |
| 2018-2021 | 9.1 | 3.4 | 6.5 | 7.2 | 6.5 | 7.2 | 7.2 | 8.4 | 11.8 | 12.2 | 11.8 | 8.7 |
| Mean | 8.7 | 5.6 | 8.3 | 8.1 | 7.6 | 8.9 | 8.4 | 8 | 8.5 | 10.3 | 9.2 | 8.3 |
| P value | 0.0481 | 0.3872 | 0.3953 | 0.9146 | 0.1334 | 0.1338 | 0.1616 | 0.5459 | 0.0658 | 0.014 | 0.709 | 0.5991 |
