# Supplementary material for: Changing trends in traumatic spinal cord injury in an aging society: Epidemiology of 1152 cases over 15 years from a single center in Japan
Source: PLoS One. 2024 May 16;19(5):e0298836. doi: 10.1371/journal.pone.0298836 (PMC11098516; doi:10.1371/journal.pone.0298836)
Supplement: Attachment — Submitted filename: PONE-D-23-30026_TC comments.pdf [file pone.0298836.s007.pdf]

PLOS ONE

Changing trends in traumatic spinal cord injury in an aging society: epidemiology of 1152 cases over 15 years from a single center in Japan

--Manuscript Draft--

Manuscript Number:	PONE-D-23-30026
Article Type:	Research Article
Full Title:	Changing trends in traumatic spinal cord injury in an aging society: epidemiology of 1152 cases over 15 years from a single center in Japan
Short Title:	Epidemiology of SCI over 15 years in Japan
Corresponding Author:	Kazuya Yokota, MD, PhD<br>Kyushu University<br>Fukuoka, JAPAN
Keywords:	spinal cord injury; epidemiology; demographics; Database; Aging society
Abstract:	Traumatic spinal cord injury (TSCI) causes an insult to the central nervous system, often resulting in devastating temporary or permanent neurological impairment and disability, which places a substantial financial burden on the health-care system. Because Japan has one of the most aging populations in the world, investigating epidemiology in this country will be significantly helpful in predicting the future global epidemiology of TSCI. This study aimed to clarify the up-to-date epidemiology and demographics of patients with TSCI treated at the largest SCI center in Japan. Data on all patients admitted to the Spinal Injuries Center with TSCI between May 2005 and December 2021 were prospectively collected using a customized, locally designed SCI database named the Japan Single Center Study for Spinal Cord Injury Database (JSSCI-DB). A total of 1152 patients were identified from the database. The study period was divided into the four- or five-year periods of 2005-2009, 2010-2013, 2014-2017, and 2018-2021 to facilitate the observation of general trends over time. The mean age of injury was 57.4 years in males and 58.1 years in females in an age range from 12 to 92 years. The age-specific analysis revealed a single peak in TSCI between 60 and 74 years. The percentage of patients 75 years of age and older increased over time to 26.2% in the 2018-2021 group. The most frequent spinal level affected by the injury was high cervical (C1-C4: 45.8%), followed by low cervical (C5-C8: 26.4%). ASIA grade A (26.6%) was the most prevalent grade among all TSCIs. The percentage of grade D TSCIs increased progressively from 2005-2009 (7.8%) to 2018-2021 (38.8%). Incomplete tetraplegia was the most common category of TSCI, accounting for 48.4% of cases. Falls on level surfaces were the most common from 2018-2021, while high or low falls were the most common injury mechanism from 2005-2009. Since 2014, the average age of injury has gradually increased to exceed 60 years. As the number of injuries among the elderly has increased, the injury mechanisms have shifted from high-fall trauma and traffic accidents to falls on level surfaces and downstairs. Incomplete tetraplegia in the elderly due to upper cervical TSCI has also increased. Our findings will be helpful in deciding treatment strategies for TSCI regarding health insurance and medical costs in future aging populations worldwide.
Order of Authors:	Kazuya Yokota, MD, PhD
	Hiroaki Sakai, MD
	Osamu Kawano, MD, PhD
	Yuichiro Morishita, MD, PhD
	Muneaki Masuda, MD, PhD
	Tetsuo Hayashi, MD, PhD
	Kensuke Kubota, MD, PhD
	Ryosuke Ideta, RPT
	Yuto Ariji, MSc, RPT
	Ryuichiro Koga, MSc, RPT
	Satoshi Murai, RPT

Powered by Editorial Manager® and ProduXion Manager® from Aries Systems Corporation

	Masatoshi Uemura, OTR
	Ryusei Ifuku, OTR
	Yasuharu Nakashima, MD, PhD
	Takeshi Maeda, MD, PhD

Additional Information:	
**Question**	**Response**
**Financial Disclosure**<br><br>Enter a financial disclosure statement that describes the sources of funding for the work included in this submission. Review the submission guidelines for detailed requirements. View published research articles from *PLOS ONE* for specific examples.<br><br>This statement is required for submission and **will appear in the published article** if the submission is accepted. Please make sure it is accurate.<br><br>**Unfunded studies**<br>Enter: *The author(s) received no specific funding for this work.*<br><br>**Funded studies**<br>Enter a statement with the following details:<br>• Initials of the authors who received each award<br>• Grant numbers awarded to each author<br>• The full name of each funder<br>• URL of each funder website<br>• Did the sponsors or funders play any role in the study design, data collection and analysis, decision to publish, or preparation of the manuscript?<br>• **NO** - Include this sentence at the end of your statement: *The funders had no role in study design, data collection and analysis, decision to publish, or preparation of the manuscript.*<br>• **YES** - Specify the role(s) played.<br><br>* typeset	The authors received no specific funding for this work.
**Competing Interests**<br><br>Use the instructions below to enter a competing interest statement for this submission. On behalf of all authors, disclose any competing interests that	The authors have declared that no competing interests exist.

could be perceived to bias this work—acknowledging all financial support and any other relevant financial or non-financial competing interests.<br><br>This statement is required for submission and **will appear in the published article** if the submission is accepted. Please make sure it is accurate and that any funding sources listed in your Funding Information later in the submission form are also declared in your Financial Disclosure statement.<br><br>View published research articles from *PLOS ONE* for specific examples.<br><br>**NO authors have competing interests**<br><br>Enter: *The authors have declared that no competing interests exist.*<br><br>**Authors with competing interests**<br><br>Enter competing interest details beginning with this statement:<br><br>*I have read the journal's policy and the authors of this manuscript have the following competing interests: [insert competing interests here]*<br><br>* typeset	
**Ethics Statement**<br><br>Enter an ethics statement for this submission. This statement is required if the study involved:<br><br>• Human participants<br>• Human specimens or tissue<br>• Vertebrate animals or cephalopods<br>• Vertebrate embryos or tissues<br>• Field research<br><br>Write "N/A" if the submission does not require an ethics statement.	This study was approved by the Ethical Review Board of Japan Labor Health and Welfare Organization Spinal Injuries Center (Approval code: 16-7). We had all the necessary consent from the patients involved in the study, including consent to participate in the study.

Powered by Editorial Manager® and ProduXion Manager® from Aries Systems Corporation

General guidance is provided below. Consult the submission guidelines for detailed instructions. **Make sure that all information entered here is included in the Methods section of the manuscript.**<br><br>**Format for specific study types**<br><br>**Human Subject Research (involving human participants and/or tissue)**<br>• Give the name of the institutional review board or ethics committee that approved the study<br>• Include the approval number and/or a statement indicating approval of this research<br>• Indicate the form of consent obtained (written/oral) or the reason that consent was not obtained (e.g. the data were analyzed anonymously)<br><br>**Animal Research (involving vertebrate animals, embryos or tissues)**<br>• Provide the name of the Institutional Animal Care and Use Committee (IACUC) or other relevant ethics board that reviewed the study protocol, and indicate whether they approved this research or granted a formal waiver of ethical approval<br>• Include an approval number if one was obtained<br>• If the study involved *non-human primates*, add *additional details* about animal welfare and steps taken to ameliorate suffering<br>• If anesthesia, euthanasia, or any kind of animal sacrifice is part of the study, include briefly which substances and/or methods were applied<br><br>**Field Research**<br><br>Include the following details if this study involves the collection of plant, animal, or other materials from a natural setting:<br>• Field permit number<br>• Name of the institution or relevant body that granted permission	
**Data Availability**<br><br>Authors are required to make all data	Yes - all data are fully available without restriction

Powered by Editorial Manager® and ProduXion Manager® from Aries Systems Corporation

underlying the findings described fully available, without restriction, and from the time of publication. PLOS allows rare exceptions to address legal and ethical concerns. See the PLOS Data Policy and FAQ for detailed information.<br><br>A Data Availability Statement describing where the data can be found is required at submission. Your answers to this question constitute the Data Availability Statement and **will be published in the article**, if accepted.<br><br>**Important:** Stating 'data available on request from the author' is not sufficient. If your data are only available upon request, select 'No' for the first question and explain your exceptional situation in the text box.<br><br>Do the authors confirm that all data underlying the findings described in their manuscript are fully available without restriction?	
**Describe where the data may be found in full sentences. If you are copying our sample text, replace any instances of XXX with the appropriate details.**<br><br>• If the data are **held or will be held in a public repository**, include URLs, accession numbers or DOIs. If this information will only be available after acceptance, indicate this by ticking the box below. For example: *All XXX files are available from the XXX database (accession number(s) XXX, XXX.).*<br>• If the data are all contained **within the manuscript and/or Supporting Information files**, enter the following: *All relevant data are within the manuscript and its Supporting Information files.*<br>• If neither of these applies but you are able to provide **details of access elsewhere**, with or without limitations, please do so. For example:<br><br>*Data cannot be shared publicly because of [XXX]. Data are available from the*	The datasets generated and/or analyzed during the current study are not publicly available from the viewpoint of personal information protection but are available from the corresponding author upon reasonable request.

XXX Institutional Data Access / Ethics Committee (contact via XXX) for researchers who meet the criteria for access to confidential data.<br><br>*The data underlying the results presented in the study are available from (include the name of the third party and contact information or URL).*<br>• This text is appropriate if the data are owned by a third party and authors do not have permission to share the data.<br><br>* typeset	
Additional data availability information:	

Powered by Editorial Manager® and ProduXion Manager® from Aries Systems Corporation

Manuscript

Click here to access/download;Manuscript;Manuscript File .docx

Changing trends in traumatic spinal cord injury in an aging society: epidemiology of 1152 cases over 15 years from a single center in Japan

Authors:

Kazuya Yokota [a, c *], Hiroaki Sakai [a], Osamu Kawano [a], Yuichiro Morishita [a], Muneaki Masuda [a], Tetsuo Hayashi [a], Kensuke Kubota [a], Ryosuke Ideta [b], Yuto Ariji [b], Ryuichiro Koga [b], Satoshi Murai [b], Ryusei Ifuku [b], Masatoshi Uemura [b], Yasuharu Nakashima [c], Takeshi Maeda [a]

Investigation performed at the Departments of Orthopaedic Surgery, Spinal Injuries Center, Fukuoka Japan

Affiliations:

Kazuya Yokota, MD, PhD

Hiroaki Sakai, MD

Osamu Kawano, MD, PhD

Yuichiro Morishita, MD, PhD

Muneaki Masuda, MD, PhD

Tetsuo Hayashi, MD, PhD

Kensuke Kubota, MD, PhD

Takeshi Maeda, MD, PhD

[a] Department of Orthopaedic Surgery, Japan Labor Health and Welfare Organization Spinal Injuries Center, 550-4 Igisu, Iizuka, Fukuoka 820-0053, Japan.

Ryosuke Ideta, RPT
Yuto Ariji, MSc, RPT
Ryuichiro Koga, MSc, RPT
Satoshi Murai, RPT
Ryusei Ifuku, OTR
Masatoshi Uemura, OTR
[b] Department of Rehabilitation Medicine, Japan Labor Health and Welfare Organization Spinal Injuries Center, 550-4 Igisu, Iizuka, Fukuoka 820-0053, Japan.

Kazuya Yokota, MD, PhD
Yasuharu Nakashima, MD, PhD
[c] Department of Orthopaedic Surgery, Graduate School of Medical Sciences, Kyushu University, 3-1-1 Maidashi, Higashiku, Fukuoka 812-8582, Japan.

[*]Address for correspondence and reprints:
Kazuya Yokota, MD, PhD
Department of Orthopaedic Surgery, Japan Labor Health and Welfare Organization Spinal Injuries Center, 550-4 Igisu, Iizuka, Fukuoka 820-0053, Japan.
Tel: +81-948-24-7500 Fax: +81-948-29-1065
E-mail: yokota.kazuya.400@m.kyushu-u.ac.jp

Abstract

Traumatic spinal cord injury (TSCI) causes an insult to the central nervous system, often resulting in devastating temporary or permanent neurological impairment and disability, which places a substantial financial burden on the health-care system. Because Japan has one of the most aging populations in the world, investigating epidemiology in this country will be significantly helpful in predicting the future global epidemiology of TSCI. This study aimed to clarify the up-to-date epidemiology and demographics of patients with TSCI treated at the largest SCI center in Japan. Data on all patients admitted to the Spinal Injuries Center with TSCI between May 2005 and December 2021 were prospectively collected using a customized, locally designed SCI database named the Japan Single Center Study for Spinal Cord Injury Database (JSSCI-DB). A total of 1152 patients were identified from the database. The study period was divided into the four- or five-year periods of 2005-2009, 2010-2013, 2014-2017, and 2018-2021 to facilitate the observation of general trends over time. The mean age of injury was 57.4 years in males and 58.1 years in females in an age range from 12 to 92 years. The age-specific analysis revealed a single peak in TSCI between 60 and 74 years. The percentage of patients 75 years of age and older increased over time to 26.2% in the 2018-2021 group. The most frequent spinal level affected by the injury was high cervical (C1-C4: 45.8%), followed by low cervical (C5-C8: 26.4%). ASIA grade A (26.6%) was the most prevalent grade among all TSCIs. The percentage of grade D TSCIs increased progressively from 2005-2009 (7.8%) to 2018-2021 (38.8%). Incomplete tetraplegia was the most common category of TSCI, accounting for 48.4% of cases. Falls on level surfaces were the most common from 2018-2021, while high or low falls were the most common injury mechanism from 2005-2009. Since 2014, the average age of injury has gradually increased to exceed 60 years. As the number of injuries among the elderly has increased, the injury mechanisms have shifted from high-fall trauma and

traffic accidents to falls on level surfaces and downstairs. Incomplete tetraplegia in the elderly due to upper cervical TSCI has also increased. Our findings will be helpful in deciding treatment strategies for TSCI regarding health insurance and medical costs in future aging populations worldwide.

Introduction

Traumatic spinal cord injury (TSCI) is an insult to the central nervous system, often resulting in devastating temporary or permanent neurological impairment and disability.[1] Historically, TSCI has been more common in young adult men and women due to injury sustained in car accidents, falls from a height, and sports.[2] Previous reports on the age distribution of TSCI showed a main peak at 10-29 years over three decades ago; however, the age of the peak group has gradually increased since the 2000s.[3, 4] The reported incidence of TSCI varies from country to country, and even from region to region within the same country.[5, 6] Recent published incidences of TSCI have ranged between 12.6 and 86 per million in the developed world.[3, 5, 7-12] In addition, recent SCIs have been characterized by an increase in cervical hyperextension injuries due to falls on level surfaces and low falls in elderly populations.[13] Understanding the up-to-date epidemiology and demographic characteristics of TSCI is crucial for managing future health care resources and identifying prevention guidelines.

According to the World Health Organization (https://www.who.int/), the global population, which increased from 3.6 billion in 1970 to 5.3 billion in 1990, exploded to 8.0 billion by 2022. By 2050, the world's population aged 60 years and older has been estimated to reach over 2.0 billion.[14] The shift in the distribution of a country's population to older age groups began in middle- and high-income countries,[14] such as Japan, where more than 30% of its population is over the age of 60.[15] According to a survey by the Japan Medical Society of Spinal Cord Lesion in 2018, the average age of TSCI patients was 66.5 years, and individuals in their 70s comprised the largest group.[11] The current aging society in Japan reflects future aging populations worldwide.[16] Hence, investigating the epidemiology of TSCI in Japan will be significantly helpful in predicting future global epidemiology of TSCI.

Approximately 60%-75% of acute TSCIs involve the cervical spine, 15% the thoracic and

10% the lumbosacral spine.[17, 18] Depending on the level and severity of injury, the costs associated with acute care, rehabilitation, and lost productivity can exceed U.S. $1 million in the first year after injury and up to U.S. $25 million over a lifetime, an annual amount of U.S. $9.7 billion.[19, 20] Understanding the epidemiology and demographics of TSCI is crucial for developing strategies to prevent future TSCIs, as well as for making decisions about post-discharge medical treatment and estimating and/or reducing health care costs. The purpose of this study is to clarify changes in the demographic and epidemiologic characteristics of individuals with TSCI over the past 15 years at a single institute in Japan based on age distribution, sex, cause of injury, level of injury, severity of injury, and seasonality in the number of patients.

Materials and Methods

The Spinal Injuries Center in Iizuka City in Fukuoka Prefecture, which opened in 1979, was originally administered by the Japanese Labor Welfare Projects Corporation.[21] The Spinal Injuries Center has a catchment area of approximately 2,000 km^2 and serves a population of approximately two million. During the 40-year period since the hospital's opening, our institution has been the only subspecialty SCI center in Japan, and almost all SCIs in Fukuoka Prefecture have been transferred to our institution for assessment and definitive treatment.

Data on all patients with TSCI admitted to the Spinal Injuries Center between May 2005 and December 2021 were collected using a customized, locally designed SCI database known as the Japan Single Center Study for Spinal Cord Injury Database (JSSCI-DB).[22, 23] We retrospectively reviewed the dataset from July to October 2022. Data were accessed for research purposes from July 1, 2022 through October 31, 2022. Only patients admitted to our institution within four weeks were included. The admitting surgeons, all of whom were fellowship-trained, dedicated spinal surgeons, and board-certified spinal surgeons approved by the board of the Japanese Society for Spine Surgery and Related Research (http://www.jssr.gr.jp/english/), were responsible for determining the patients' diagnoses, neurology, and treatment strategies. The data were compiled by spinal research coordinators and peer-reviewed by other board-certified spinal surgeons at weekly meetings for accuracy and completeness. Patients with neurological deficits secondary to nontraumatic conditions, such as tumors, infections, vascular abnormalities, and psychogenic paralysis, were excluded.

The demographic data collected prospectively included age, sex, height, weight, date of injury, mechanism of injury, length of hospital stay, American Spinal Injury Association (ASIA) Impairment Scale (AIS), spinal or neurological level of injury (NLI), details of treatment, and alcohol consumption at injury. The International Standard for the Neurological Classification

of Spinal Cord Injury (ISNCSCI) examinations were conducted by physicians and physician assistants who had completed the ASIA International Standards Training e-Learning Program (InSTeP) as well as in-person training. The mechanism of injury was classified as a fall on a level surface, fall downstairs, traffic accident by car, traffic accident by motorcycle, traffic accident by bicycle, fall from ≥ 3m (high fall), fall from < 3 m (low fall), struck by object, sports, and unspecified.

The study period was divided into four- and five-year groups to facilitate the observation of general trends over time depending on the year of the occurrence of the injury: 2005-2009, 2010-2013, 2014-2017, and 2018-2021. Registration in the JSSCI-DB began in the middle of 2005. During the period from May 2011 to December 2011, patient data could not be entered into the database because of hospital renovations; therefore, the number of registered patients decreased considerably in 2005 and 2011. To describe the age groups in this population, we established the following six age ranges: 0-14, 15-29, 30-44, 45-59, 60-74, and more than 75 years.[3] The affected neurological levels were classified as C1-C4, C5-C8, T1-T6, T7-T12, and L1-L5. In addition, the AIS grades of the SCI were categorized as complete or incomplete tetraplegia and complete or incomplete paraplegia.

Statistical Analysis

All statistical analyses were performed using the JMP software program version 13 (SAS Institute, Cary, NC, USA). Descriptive statistics were reported using proportions for the categorical data and means for the continuous data. Trends and relationships over the 15-year study period were examined for strength of association using the chi-square test. In all statistical analyses, significance was defined as $P < 0.05$.

Results

A total of 1152 patients were identified from our database over the 15-year study period from May 2005 to December 2021. A summary of the data on this population is shown in Tables 1 and 2. The absolute number of cases per year ranged from 61 (in 2019) to 88 (in 2014), with a mean of 70 cases, except in 2005 and 2011 (Figure 1A). The percentages of injured patients who were male ranged from 76.4% (in 2021) to 88.5% (in 2008), with a mean of 81.5% (Figure 1B). The mean age of injury was 57.4 years in males and 58.1 years in females, with an age range from 12-92 years (Figure 1C). The age-specific analysis revealed a single peak in TSCI between 60 and 74 years. From 2005-2009, the 45-59 age group was the second most common, but since 2010, the 75+ age group was the second most common group after the 60-74 age group. The percentage of patients 75 years and older increased over time, comprising 26.2% of the patients in the 2018-2021 group (Figure 1D and Table 2). The most frequent spinal level affected by the injury was high cervical (C1-C4: 45.8%), followed by low cervical (C5-C8: 26.4%), lumbar (L1-L5: 13.3%), high thoracic (T1-T6: 3.1%), and low thoracic (T7-T12: 11.3%). Throughout the 2005-2021 period, cervical TSCI accounted for 70% or more of all TSCI patients (Figure 1E, Tables 1 and 3).

Regarding the extent of injury, 26.6% of the cases were classified as ASIA grade A, 10.2% as grade B, 25.9% as grade C, 22.4% as grade D, and 14.8% as grade E. ASIA grade A was the most prevalent in all TSCIs. Notably, the percentage of grade D TSCIs increased dramatically from 2005-2009 (7.8%) to 2018-2021 (38.8%) ($P < 0.0001$). Incomplete tetraplegia was the most common category of SCI, accounting for 48.4% of the cases (Table 1). The percentage of SCIs as incomplete tetraplegia also increased progressively from 2005-2009 (43.7%) to 2018-2021 (52.1%) ($P < 0.0001$), while the percentage of SCIs as complete paraplegia decreased from 2005-2009 (11.7%) to 2018-2021 (4.2%) (Table 3).

The operation rate of cases was 52.17% throughout the study period, with the highest percentage of operation rate in 2018-2021 (59.7%) (Tables 1 and 2). The average length of stay in our institution was 181.6 (s.d. ± 146.8) days, and the average time to admission after SCI was 3.16 (s.d. ± 5.14) days (Table 1).

The most frequent causes of SCI during the study period were falls (62.2%), followed by traffic accidents (24.5%), struck by objects (8.2%), and sports (4.9%) (Table 1). The analysis of injury mechanisms revealed that falls on a level surface were the most common in 2018-2021, while high or low falls were the most common injury mechanism from 2005-2009 (Table 3). Regarding the characteristics of the etiological distribution of the injuries over the study period, the most relevant changes were an increase in the number of falls on level surfaces and downstairs, and a decrease in the number of falls during sports (Table 3). The percentage of injured patients with an etiology linked to alcohol use ranged from 13.2% (2005-2008) to 19% (2014-2017), with a mean of 16.1% (Table 3).

The peak days for the occurrence of SCIs were Friday, Saturday, and Sunday. Throughout the entire 2005-2021 period, there were more than 150 cases on each day of the week, with no significant differences by day of the week (Figure 2A and Supplemental Tables 1 and 2) ($P = 0.6136$). Seasonal variations in SCI incidences differed from year to year, and SCIs occurred more often during the fall months and least frequently in the winter months. SCIs were the most common in October and the least common in February (Figures 2B and 2C, Supplemental Tables 3 and 4) ($P = 0.0666$).

Discussion

In this study, we conducted a detailed investigation of the epidemiology and demographics of TSCI over a period of 15 years at a single institution in Japan. The mean age at injury increased over time, demonstrating an increasing trend in the proportion of elderly TSCIs over 60 years. A characteristic feature of TSCI in the elderly was that the injury was at the cervical level (C1-C4 or C5-C8), and the severity of injury included a comparatively large portion of TSCIs with mild neurological impairment, such as AIS C or D.

The mean age at injury in the most recent period from 2018-2021 was 60 years, whereas the mean age was 66.5 years in a Japanese nationwide epidemiological study conducted in 2018.[11] Furthermore, the percentage of falls on level surfaces accounted for 18.6% in this study, corresponding to about one-half of the number (38.6%) in the nationwide study. Because our facility is actively involved in the social reintegration process after SCI in younger patients,[24] the proportion of patients with TSCI due to traffic trauma and sports injuries remained high, leading to a decrease in the proportion of elderly patients with TSCI in our database. Cervical SCI in the elderly increases the likelihood of developing complications, such as pneumonia, urinary tract infection, and decubitus ulcers,[25] which may imply that elderly cases could not receive satisfactory treatment in specialized facilities such as ours because of the priority given to the systemic management of the treatment of such complications. This study is one of the few to accurately show the percentage of TSCIs in patients who were consuming alcohol at the time of injury.[26] The findings showed that 13.2% to 19% of the patients had consumed alcohol. Avoiding excessive alcohol consumption may help prevent the occurrence of SCIs in the elderly. Because populations worldwide are aging, the recent increase in cervical SCI in the elderly will be an important issue that could accelerate the pressure on health care costs in many countries, including currently developing countries.[27]

This epidemiologic study was based on prospectively collected data on more than 1000 patients with acute TSCI who were admitted to our institution, which is the largest reported population-based sample of TSCI in single institution in Japan. The single-institution database has advantages over multicenter studies. First, all examiners who collected neurological findings in this study had completed formal InSTeP training, and the decided AIS grades and NLIs were subsequently reviewed by similarly qualified colleagues, which strongly ensured the quality of this database. In collecting neurological data on TSCI in a multicenter study, because previous assessors may not have completed formal examination training, they would have been less reliable in determining neurological outcomes. Indeed, Kirshblum and colleagues presented challenging classifications along with responses and explanations,[28, 29] even though the ISNCSCI endorsed by ASIA and the International Spinal Cord Society (ISCoS) has undergone revision and updates over time.[30] For instance, in some challenging cases, patients have been diagnosed with AIS A due to the lack of sacral sparing, although from the standpoint of walking ability, the patients appear to have AIS D-like capability because muscle strength was significantly preserved.[31] Second, we prospectively entered the neurological findings of all patients in electronic medical records and determined both NLI and AIS grades according to the exact diagnostic criteria officially sanctioned by ASIA. According to Miyakoshi and colleagues, the nationwide survey was based on a retrospective design, which may have resulted in missing values and an insufficient amount of data. Integrating our database system into core spinal centers across the country will lead to increasing the number and accuracy of nationwide epidemiological studies of TSCI. A previous report suggested inaccuracies in the manual ISNCSCI worksheets in the clinical setting and 75% of ISNCSCI worksheets had one or more errors when completed manually while undertaking randomized controlled trials.[32] Osunronbi and colleagues indicated that the quality of ISNSCI

documentation remained poor, and they proposed that reasons for the low quality of ISNSCI documentation included negligence and lack of knowledge.[33] Well-established training and a computerized algorithm may be necessary to ensure accurate scoring, scaling, and classification of the ISNSCI. Third, our facility is unique because after incorporating them into our database from the acute stage of SCI, we were able to follow up patients in the same institution over a long period. The average length of hospital stay was more than 180 days. The average time of admission of the patients shown in this study was 3.16 days (0-28 days) after injury. In contrast, most previous demographic studies did not specify the time to hospitalization after injury. We believe that our facility will play a critical role in future clinical trials in Japan, as it can provide long-term follow-up data on acute operative and non-operative care, as well as chronic rehabilitation care following SCI.

This study has several limitations. First, because the registration of the database began in the middle of 2005, and the hospital was renovated in 2011, the data for these years are not complete. Second, the patients' hospitalization periods ranged from 0-28 days after their injury. Within four weeks of an injury, the AIS grade could change significantly, and even small differences in the time of evaluation could affect the neurological findings on admission. Third, our facility did not deal with pediatric trauma cases under 14 years of age. The very small number of pediatric TSCI cases may have resulted in an increase in the average age at the time of injury. Finally, the data in this study included information under patent, and not all of the data could be made publicly available. Despite these limitations, our results were derived from the only database in Japan that has been complied in detail at a single institution. Therefore, we believe that our database and the results of the present study will aid in developing future strategies for treating SCI in aging populations.

Conclusion

This study presents the prospective findings of the demographics and epidemiology of TSCI in Japan over a period of more than 15 years since 2005. The incidence of TSCI was consistently higher in men than in women. Throughout the study period, approximately 80% of the patients were. Since 2014, the average age at injury has gradually increasing to exceed 60 years. As the number of injuries among the elderly increased, the injury mechanisms shifted from high-fall trauma and traffic accidents to falls on level surfaces and downstairs. Incomplete tetraplegia in the elderly due to upper cervical SCI also increased. Falls while consuming alcohol may also have significantly impacted the occurrence of SCI. To accurately determine the epidemiology of SCI patients, it is necessary to establish an environment that allows for the long-term consistent follow up of patients in specialized facilities by professional staff. Because Japan has one of the highest aging populations in the world, epidemiological studies in this country will be very helpful in determining health insurance and medical costs and deciding treatment strategies for SCI in future aging populations worldwide.

References

1. Ahuja CS, Wilson JR, Nori S, Kotter MRN, Druschel C, Curt A, et al. Traumatic spinal cord injury. Nature reviews Disease primers. 2017;3:17018. Epub 2017/04/28. doi: 10.1038/nrdp.2017.18. PubMed PMID: 28447605.

2. Shingu H, Ohama M, Ikata T, Katoh S, Akatsu T. A nationwide epidemiological survey of spinal cord injuries in Japan from January 1990 to December 1992. Paraplegia. 1995;33(4):183-8. Epub 1995/04/01. doi: 10.1038/sc.1995.42. PubMed PMID: 7609973.

3. Chen Y, He Y, DeVivo MJ. Changing Demographics and Injury Profile of New Traumatic Spinal Cord Injuries in the United States, 1972-2014. Archives of physical medicine and rehabilitation. 2016;97(10):1610-9. Epub 2016/04/26. doi: 10.1016/j.apmr.2016.03.017. PubMed PMID: 27109331.

4. Jain NB, Ayers GD, Peterson EN, Harris MB, Morse L, O'Connor KC, et al. Traumatic spinal cord injury in the United States, 1993-2012. Jama. 2015;313(22):2236-43. Epub 2015/06/10. doi: 10.1001/jama.2015.6250. PubMed PMID: 26057284; PubMed Central PMCID: PMCPMC4712685.

5. Singh A, Tetreault L, Kalsi-Ryan S, Nouri A, Fehlings MG. Global prevalence and incidence of traumatic spinal cord injury. Clinical epidemiology. 2014;6:309-31. Epub 2014/10/04. doi: 10.2147/clep.s68889. PubMed PMID: 25278785; PubMed Central PMCID: PMCPMC4179833.

6. Ding W, Hu S, Wang P, Kang H, Peng R, Dong Y, et al. Spinal Cord Injury: The Global Incidence, Prevalence, and Disability From the Global Burden of Disease Study 2019. Spine. 2022;47(21):1532-40. Epub 2022/07/21. doi: 10.1097/brs.0000000000004417. PubMed PMID: 35857624; PubMed Central PMCID: PMCPMC9554757.

7. Halvorsen A, Pettersen AL, Nilsen SM, Halle KK, Schaanning EE, Rekand T.

Epidemiology of traumatic spinal cord injury in Norway in 2012-2016: a registry-based cross-sectional study. Spinal cord. 2019;57(4):331-8. Epub 2018/12/24. doi: 10.1038/s41393-018-0225-5. PubMed PMID: 30573770.

8. Ferro S, Cecconi L, Bonavita J, Pagliacci MC, Biggeri A, Franceschini M. Incidence of traumatic spinal cord injury in Italy during 2013-2014: a population-based study. Spinal cord. 2017;55(12):1103-7. Epub 2017/09/06. doi: 10.1038/sc.2017.88. PubMed PMID: 28872148.

9. Beck B, Cameron PA, Braaf S, Nunn A, Fitzgerald MC, Judson RT, et al. Traumatic spinal cord injury in Victoria, 2007-2016. The Medical journal of Australia. 2019;210(8):360-6. Epub 2019/05/06. doi: 10.5694/mja2.50143. PubMed PMID: 31055854.

10. Kudo D, Miyakoshi N, Hongo M, Kasukawa Y, Ishikawa Y, Ishikawa N, et al. An epidemiological study of traumatic spinal cord injuries in the fastest aging area in Japan. Spinal cord. 2019;57(6):509-15. Epub 2019/02/09. doi: 10.1038/s41393-019-0255-7. PubMed PMID: 30733575.

11. Miyakoshi N, Suda K, Kudo D, Sakai H, Nakagawa Y, Mikami Y, et al. A nationwide survey on the incidence and characteristics of traumatic spinal cord injury in Japan in 2018. Spinal cord. 2021;59(6):626-34. Epub 2020/08/13. doi: 10.1038/s41393-020-00533-0. PubMed PMID: 32782342.

12. Lenehan B, Street J, Kwon BK, Noonan V, Zhang H, Fisher CG, et al. The epidemiology of traumatic spinal cord injury in British Columbia, Canada. Spine. 2012;37(4):321-9. Epub 2012/02/18. doi: 10.1097/BRS.0b013e31822e5ff8. PubMed PMID: 22337075.

13. Morishita Y, Maeda T, Naito M, Ueta T, Shiba K. The pincers effect on cervical spinal cord in the development of traumatic cervical spinal cord injury without major fracture or dislocation. Spinal cord. 2013;51(4):331-3. Epub 2012/12/05. doi: 10.1038/sc.2012.157.

PubMed PMID: 23208542.

14. Xi JY, Lin X, Hao YT. Measurement and projection of the burden of disease attributable to population aging in 188 countries, 1990-2050: A population-based study. Journal of global health. 2022;12:04093. Epub 2022/10/20. doi: 10.7189/jogh.12.04093. PubMed PMID: 36259226; PubMed Central PMCID: PMCPMC9579832 Interest form (available upon request from the corresponding author), and declare no conflicts of interest.

15. Nakatani H. Population aging in Japan: policy transformation, sustainable development goals, universal health coverage, and social determinates of health. Global health & medicine. 2019;1(1):3-10. Epub 2019/10/31. doi: 10.35772/ghm.2019.01011. PubMed PMID: 33330747; PubMed Central PMCID: PMCPMC7731274.

16. Chang AY, Skirbekk VF, Tyrovolas S, Kassebaum NJ, Dieleman JL. Measuring population ageing: an analysis of the Global Burden of Disease Study 2017. The Lancet Public health. 2019;4(3):e159-e67. Epub 2019/03/11. doi: 10.1016/s2468-2667(19)30019-2. PubMed PMID: 30851869; PubMed Central PMCID: PMCPMC6472541.

17. Ackery A, Tator C, Krassioukov A. A global perspective on spinal cord injury epidemiology. Journal of neurotrauma. 2004;21(10):1355-70. Epub 2005/01/28. doi: 10.1089/neu.2004.21.1355. PubMed PMID: 15672627.

18. Sekhon LH, Fehlings MG. Epidemiology, demographics, and pathophysiology of acute spinal cord injury. Spine. 2001;26(24 Suppl):S2-12. Epub 2002/01/24. doi: 10.1097/00007632-200112151-00002. PubMed PMID: 11805601.

19. Spinal Cord Injury (SCI) 2016 Facts and Figures at a Glance. The journal of spinal cord medicine. 2016;39(4):493-4. Epub 2016/07/30. doi: 10.1080/10790268.2016.1210925. PubMed PMID: 27471859; PubMed Central PMCID: PMCPMC5102286.

20. Merritt CH, Taylor MA, Yelton CJ, Ray SK. Economic impact of traumatic spinal cord

injuries in the United States. Neuroimmunology and neuroinflammation. 2019;6. Epub 2019/01/01. doi: 10.20517/2347-8659.2019.15. PubMed PMID: 33869674; PubMed Central PMCID: PMCPMC8052100.

21. Akatsu T. A new Spinal Injuries Centre at Iizuka City in Japan. Paraplegia. 1982;20(5):313-4. Epub 1982/10/01. doi: 10.1038/sc.1982.59. PubMed PMID: 7177699.

22. Ariji Y, Hayashi T, Ideta R, Koga R, Murai S, Towatari F, et al. A prediction model of functional outcome at 6 months using clinical findings of a person with traumatic spinal cord injury at 1 month after injury. Spinal cord. 2020;58(11):1158-65. Epub 2020/05/24. doi: 10.1038/s41393-020-0488-5. PubMed PMID: 32444638.

23. Ariji Y, Hayashi T, Ideta R, Koga R, Murai S, Naka T, et al. Identification of a reliable sacral-sparing examination to assess the ASIA impairment scale in patients with traumatic spinal cord injury. The journal of spinal cord medicine. 2022:1-7. Epub 2022/03/31. doi: 10.1080/10790268.2022.2047548. PubMed PMID: 35352975.

24. Iwatsubo E, Shiba K, Ueta T, Takemitsu Y, Akatsu T. A report on the development and work of the Labor Welfare Corporation Spinal Injuries Center in Japan. Spinal cord. 1997;35(11):718-9. Epub 1997/12/10. doi: 10.1038/sj.sc.3100544. PubMed PMID: 9392039.

25. Yamashita M, Nagata K, Takami M, Okada M, Takiguchi N, Enyo Y, et al. Mortality and complications in elderly patients with cervical spine injuries. Injury. 2022;53(6):2114-20. Epub 2022/05/07. doi: 10.1016/j.injury.2022.04.015. PubMed PMID: 35513939.

26. Mirzaeva L, Gilhus NE, Lobzin S, Rekand T. Incidence of adult traumatic spinal cord injury in Saint Petersburg, Russia. Spinal cord. 2019;57(8):692-9. Epub 2019/03/08. doi: 10.1038/s41393-019-0266-4. PubMed PMID: 30842631.

27. Furlan JC, Fehlings MG, Craven BC. Economic Impact of Aging on the Initial Spine Care of Patients With Acute Spine Trauma: From Bedside to Teller. Neurosurgery.

2019;84(6):1251-60. Epub 2018/05/24. doi: 10.1093/neuros/nyy180. PubMed PMID: 29790981.

28. Kirshblum S, Schmidt Read M, Rupp R. Classification challenges of the 2019 revised International Standards for Neurological Classification of Spinal Cord Injury (ISNCSCI). Spinal cord. 2022;60(1):11-7. Epub 2021/06/06. doi: 10.1038/s41393-021-00648-y. PubMed PMID: 34088981; PubMed Central PMCID: PMCPMC8737267.

29. Kirshblum SC, Biering-Sorensen F, Betz R, Burns S, Donovan W, Graves DE, et al. International Standards for Neurological Classification of Spinal Cord Injury: cases with classification challenges. The journal of spinal cord medicine. 2014;37(2):120-7. Epub 2014/02/25. doi: 10.1179/2045772314y.0000000196. PubMed PMID: 24559416; PubMed Central PMCID: PMCPMC4066420.

30. Rupp R, Biering-Sørensen F, Burns SP, Graves DE, Guest J, Jones L, et al. International Standards for Neurological Classification of Spinal Cord Injury: Revised 2019. Topics in spinal cord injury rehabilitation. 2021;27(2):1-22. Epub 2021/06/11. doi: 10.46292/sci2702-1. PubMed PMID: 34108832; PubMed Central PMCID: PMCPMC8152171.

31. Zariffa J, Curt A, Steeves JD. Functional motor preservation below the level of injury in subjects with American Spinal Injury Association Impairment Scale grade A spinal cord injuries. Archives of physical medicine and rehabilitation. 2012;93(5):905-7. Epub 2012/03/01. doi: 10.1016/j.apmr.2011.10.024. PubMed PMID: 22360976.

32. Armstrong AJ, Clark JM, Ho DT, Payne CJ, Nolan S, Goodes LM, et al. Achieving assessor accuracy on the International Standards for Neurological Classification of Spinal Cord Injury. Spinal cord. 2017;55(11):994-1001. Epub 2017/06/21. doi: 10.1038/sc.2017.67. PubMed PMID: 28631745.

33. Osunronbi T, Sharma H. International Standards for Neurological Classification of

Spinal Cord Injury: factors influencing the frequency, completion and accuracy of documentation of neurology for patients with traumatic spinal cord injuries. European journal of orthopaedic surgery & traumatology : orthopedie traumatologie. 2019;29(8):1639-48. Epub 2019/07/22. doi: 10.1007/s00590-019-02502-7. PubMed PMID: 31324967; PubMed Central PMCID: PMCPMC6851215.

Declarations

Ethics approval and consent to participate

This study was approved by the Ethical Review Board of Japan Labor Health and Welfare Organization Spinal Injuries Center (Approval code: 16-7). We had all the necessary consent from the patients involved in the study, including consent to participate in the study.

Consent for publication

Written informed consent for publication of their clinical details was obtained for the patients.

Data availability

The datasets generated and/or analyzed during the current study are not publicly available from the viewpoint of personal information protection but are available from the corresponding author upon reasonable request.

Funding

This study was supported by JSPS KAKENHI Grant Number 21K16671.

Authors' contributions

Kazuya Yokota designed the study and analyzed the datasets with technical help from Hiroaki Sakai. Kazuya Yokota, Hiroaki Sakai, Osamu Kawano, Yuichiro Morishita, Muneaki Masuda, Tetsuo Hayashi, Kensuke Kubota, Ryosuke Ideta, Yuto Ariji, Ryuichiro Koga, Satoshi Murai, Ryusei Ifuku and Masatoshi Uemura collected the patients' data. Yasuharu Nakashima and Takeshi Maeda provided technical support and ideas for the project. Kazuya Yokota supervised the overall project and performed the final manuscript preparation. All authors read and

approved the final manuscript.

Acknowledgements

Not applicable.

Conflict of Interest

The authors declare that they have no conflict of interest.

List of abbreviations

ASIA: American Spinal Injury Association; AIS: ASIA Impairment Scale; NLI: neurological level of injury; TSCI: traumatic spinal cord injury; JSSCI-DB: Japan Single Center study for Spinal Cord Injury Database; InSTeP: International Standards Training e-Learning Program; ISNCSCI: International Standard for Neurological Classification of Spina Cord Injury

Figure Legends

Figure 1.

(A and B) Number (A) and percentage (B) of traumatic spinal cord injuries (TSCI) from 2005 to 2021 (n = 1152). The incidence rate was consistently higher in men than in women. Registration at the Japan Single Center Study for Spinal Cord Injury Database (JSSCI-DB) began in the middle of 2005. Patient data could not be entered into the database because of hospital renovations from May 2011 to December 2011; therefore, the number of registered patients decreased considerably in 2005 and 2011.

(C) Temporal change in average age at injury, with a slight stepwise increase from 2005 to 2021.

(D and [illegible] Percentages of TSCI by age group from 2005 to 2021. The 60-74 age group was the most common group, and the 75+ age group was the second most common, beginning in 2010.

(E) Percentages of TSCI by neurological level of injury (NLI) from 2005 to 2021. The most frequent NLI affected by the injury was the high cervical level (C1-C4), followed by the low cervical level (C5-C8).

Figure 2.

(A) Weekday variations in injuries and the number of TSCI cases per weekday (n = 1152). The peak days for the occurrence of TSCI were Friday, Saturday, and Sunday. Throughout the entire period, there were more than 150 cases each day of the week, with no significant differences according to the day of the week.

(B and C) Seasonal variations in injuries and the number of TSCI cases per month. TSCIs were most common in October and least common in February. TSCIs occurred more often during the fall months and were the least common in the winter months.

Table 1. Overall traumatic spinal cord injury demographics (2005-2021, total = 1152)

Age at injury (year)	Male: 57.4 ± 18.7, Female: 58.1 ± 22.0
Sex (n, %)	Male: 935 (81.2), Female: 217 (18.8)
Height (cm), Weight (kg), BMI	164.8 ± 8.65, 61.1 ± 11.9, 22.4 ± 3.54
Level of injury (n, %)	C1-C4: 528 (45.8), C5-C8: 304 (26.4), T1-T6: 36 (3.1), T7-T12: 131 (11.3), L1-L5: 153 (13.3)
Mechanism of injury (n, %)	Fall: 717 (62.2), Traffic accident: 282 (24.5), Sports: 57 (4.9), Struck by object: 94 (8.2), Unknown: 2 (0.2)
ASIA Impairment Scale at admission (n, %)	A: 307 (26.6), B: 118 (10.2), C: 298 (25.9), D: 258 (22.4), E: 171 (14.8)
Tetraplegia (n, %)	Complete: 219 (19), Incomplete: 558 (48.4)
Operation rate (n, %)	601 (52.17)
Days to admission after SCI	3.16 ± 5.14 (0-28), IQR = (0-3)
Length of stay (days)	181.6 ± 146.8 (1-1067), IQR = (55-286)

ASIA: American Spinal Injury Association; IQR: interquartile range; Variables are given as the mean and standard deviation with the range in parenthesis or as the number with the percentage in parenthesis.

Table 2. Demographic profile of patients with TSCI

	2005-2009	2010-2013	2014-2017	2018-2021
Sample size (n)	334	250	305	263
Age at injury	52.6 ± 20.3	58.4 ± 19.6	60.2 ± 17.7	60. 0 ± 18.7
Age group				
0 – 14	2 (0.6)	1 (0.4)	0 (0)	1 (0.4)
15 – 29	60 (18)	32 (12.8)	26 (8.5)	24 (9.1)
30 – 44	45 (13.5)	30 (12)	28 (9.2)	30 (11.4)
45 – 59	75 (22.5)	29 (11.6)	49 (16.1)	43 (16.3)
60 – 74	96 (28.7)	102 (40.8)	125 (41)	96 (36.5)
75+	56 (16.8)	56 (22.4)	77 (25.2)	69 (26.2)
Male/Female	278 (83.2)/56	203 (81.2)/47	241 (79)/64	213 (81)/50
Height (cm)	164.6 ± 8.71	164.2 ± 8.1	164.8 ± 8.9	165.4 ± 8.78
Weight (kg)	61.4 ± 11.3	60.7 ± 12.2	59.9 ± 11.5	62.7 ± 12.7
BMI (kg/m^2)	22.6 ± 3.25	22.4 ± 3.67	22 ± 3.51	22.8 ± 3.76
Operation rate	183 (54.8)	116 (46.4)	145 (47.5)	157 (59.7)

Variables are given as the mean and standard deviation with the range in parenthesis or as the number with the percentage in parenthesis.

Table 3. Injury profile of patients with TSCI

Characteristics	Injury Year Intervals			
	2005-2009	2010-2013	2014-2017	2018-2021
Sample size (n)	334	250	305	263
Etiology of injury				
Fall on level surface (n, %)	44 (13.2)	37 (14.8)	68 (22.3)	49 (18.6)
Fall downstairs (n, %)	18 (5.1)	22 (8.8)	26 (8.5)	33 (12.5)
Traffic accident car (n, %)	39 (11.7)	49 (19.6)	34 (11.1)	28 (10.6)
Traffic accident motorcycle (n, %)	28 (8.4)	9 (3.6)	24 (7.9)	12 (4.6)
Traffic accident bicycle (n, %)	16 (4.8)	16 (6.4)	9 (3)	18 (6.8)
High fall (n, %)	56 (16.8)	43 (17.2)	42 (13.8)	43 (16.3)
Low fall (n, %)	79 (23.7)	40 (16)	71 (23.3)	46 (17.5)
Struck by object (n, %)	30 (9)	21 (8.4)	21 (6.9)	22 (8.4)
Sports (n, %)	24 (7.2)	13 (5.2)	9 (3)	11 (4.2)
Unspecified or unknown (n, %)	0 (0)	0 (0)	1 (0.3)	1 (0.4)
Alcohol consumption at injury				
Yes	44 (13.2)	41 (16.4)	58 (19)	42 (16)
No	290 (86.8)	207 (82.8)	240 (78.7)	214 (81.4)
Unspecified or unknown	0 (0)	2 (0.8)	7 (2.3)	7 (2.7)
Neurological level of injury				
C1–C4	123 (45.2)	117 (52.5)	147 (55.1)	105 (47.9)
C5–C8	82 (30.1)	59 (26.5)	74 (27.7)	70 (32.0)
T1–T6	11 (4)	8 (3.6)	4 (1.5)	5 (2.3)

T7–T12	29 (10.7)	31 (13.9)	26 (9.7)	15 (6.8)
L1–L5	27 (9.9)	8 (3.6)	16 (6)	24 (11)
AIS/Frankel grade				
A	98 (29.3)	77 (30.8)	83 (27.2)	49 (18.6)
B	36 (10.8)	28 (11.2)	29 (9.5)	25 (9.5)
C	112 (33.5)	67 (26.8)	76 (24.9)	43 (16.3)
D	26 (7.8)	51 (20.4)	79 (25.9)	102 (38.8)
E	62 (18.6)	27 (10.8)	38 (12.5)	44 (16.7)
Category of SCI				
Complete tetraplegia	59 (17.7)	57 (22.8)	65 (21.3)	38 (14.4)
Incomplete tetraplegia	146 (43.7)	119 (47.6)	156 (51.1)	137 (52.1)
Complete paraplegia	39 (11.7)	20 (8)	18 (5.9)	11 (4.2)
Incomplete paraplegia	28 (8.4)	27 (10.8)	28 (9.2)	33 (12.5)

Variables are given as the mean with the percentage in parenthesis.

Click here to access/download;Figure;Figure 1.eps

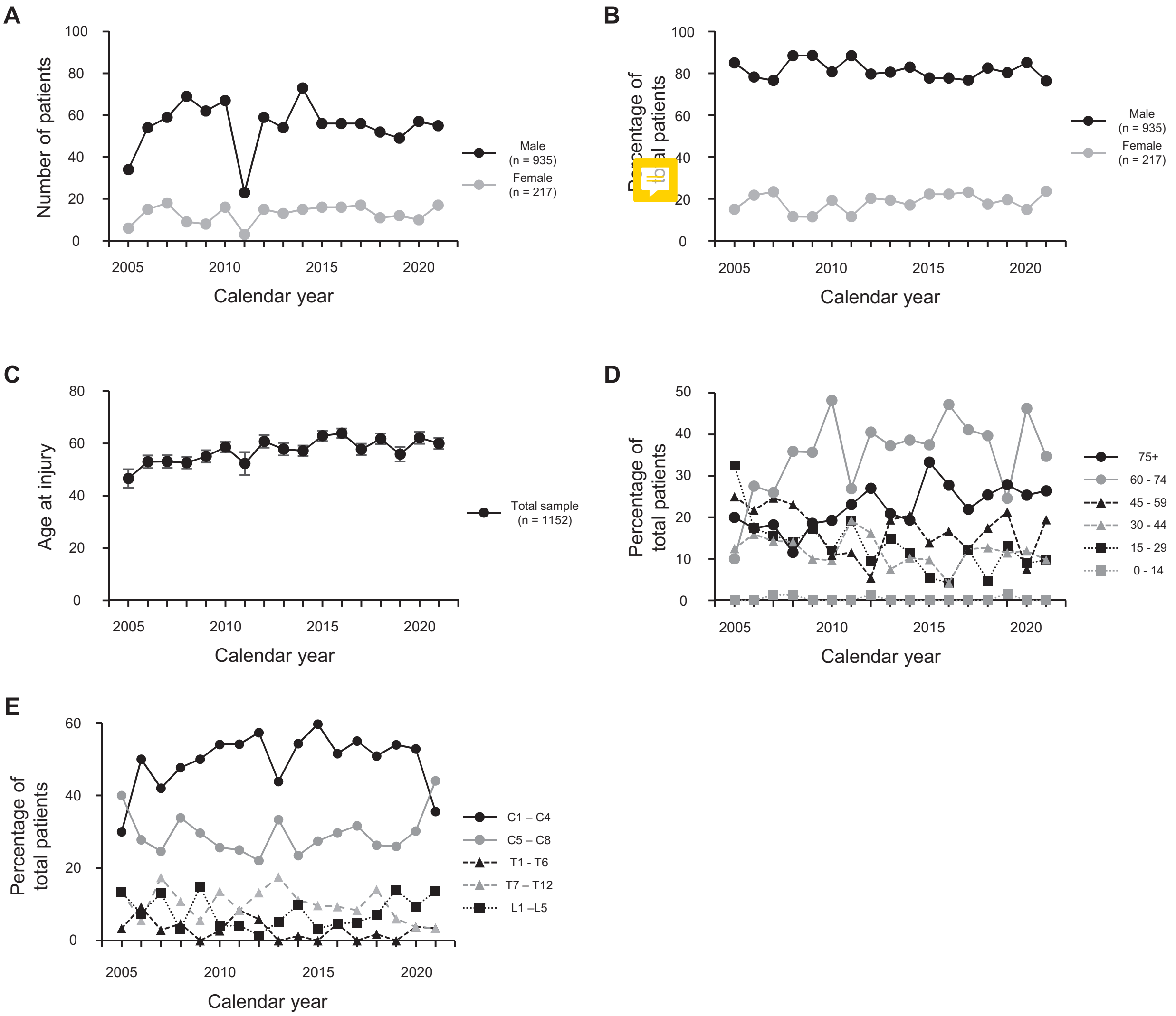

Figure 1

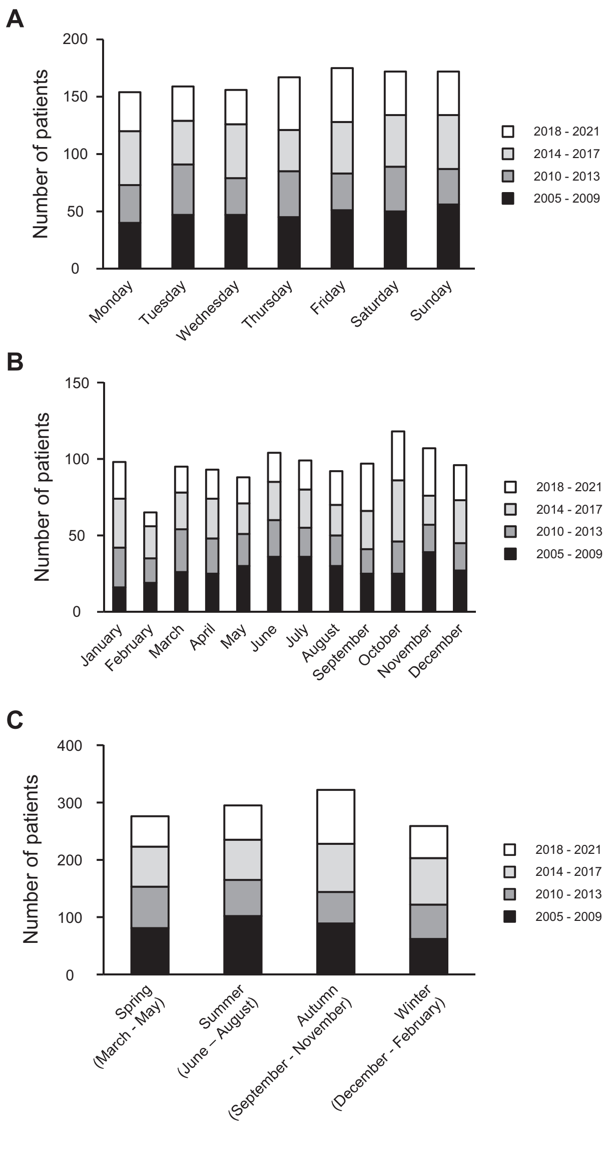
A
Number of patients
0
50
100
150
200
Monday
Tuesday
Wednesday
Thursday
Friday
Saturday
Sunday
2018 - 2021
2014 - 2017
2010 - 2013
2005 - 2009
B
Number of patients
0
50
100
150
January
February
March
April
May
June
July
August
September
October
November
December
2018 - 2021
2014 - 2017
2010 - 2013
2005 - 2009
C
Number of patients
0
100
200
300
400
Spring (March - May)
Summer (June – August)
Autumn (September - November)
Winter (December - February)
2018 - 2021
2014 - 2017
2010 - 2013
2005 - 2009

Figure 2

Click here to access/download
Supporting Information
Supplementary Tables.docx

Click here to access/download
Supporting Information
Proofreading Certificate.pdf

Click here to access/download
Supporting Information
Human Participants Research Checklist.docx

Click here to access/download
Supporting Information
Original Ethical Approval Document.pdf

Click here to access/download
Supporting Information
Translated Ethical Approval Document.pdf
