# Supplementary material for: Changing trends in traumatic spinal cord injury in an aging society: Epidemiology of 1152 cases over 15 years from a single center in Japan
Source: PLoS One. 2024 May 16;19(5):e0298836. doi: 10.1371/journal.pone.0298836 (PMC11098516; doi:10.1371/journal.pone.0298836)
Supplement: Attachment — Submitted filename: Response to Reviewers.docx [file pone.0298836.s008.docx]

To the editor and reviewers:

We appreciate the reviewer’s constructive comments that significantly improved the quality of the manuscript. We have addressed each reviewer’s comments point-by-point and would appreciate the opportunity to further clarify any questions that remain after this round of revisions. The reviewers’ comments are shown in **bold face** and are followed by our responses. The changes made in the revised manuscript are indicated in underlined italic text.

**Reviewer(s)' Comments to Author:**

**Reviewer #1: The authors performed a study addressing “Changing trends in traumatic spinal cord injury in an aging society: epidemiology of 1152 cases over 15 years from a single center in Japan”. However, there remain several concerns to be clarified, some of which are critical.**

We thank Reviewer 1 for the critical evaluation of the manuscript. By addressing the reasonable and insightful comments from the reviewer, the manuscript has been significantly improved.

**1. It is unclear what you want to see in this study. “The purpose of this study is to clarify changes in the demographic and epidemiologic characteristics of individuals with TSCI over the past 15 years based on age in a single institute in Japan. changes in the demographic and epidemiologic characteristics of individuals with TSCI over the past 15 years at a single institute in Japan based on age distribution, sex, cause of injury, level of injury, severity of injury, and seasonality in the number of patients.” (p.6 line 6-10) The purpose is not clear in the results of this study. Please correct it.**

As highlighted by the reviewer, we recognize that the lack of clarity in the study's objectives diminishes the value of this paper. Formulating a specific and clear research objective within the context of a general epidemiological research paper can be challenging. Despite this, we have worked to enhance the clarity of our research objective, modifying the introduction to make it more easily understandable.

*Page 7 line 7 in the Introduction section*

*The purpose of this study is to elucidate the current epidemiology and demographics of patients with TSCIs at the largest SCI center in Japan over the past 15 years. Additionally, the study aims to propose future strategies for the prevention of TSCIs.*

**2. Please describe the results (including tables and figures), including the site of injury and cause of injury by age group.**

In accordance with the reviewer's instructions, we generated a table detailing the site of injury and cause of injury by age group.

*Table 4.*

After consulting statistical experts, we opted for the Cochran-Armitage test to analyze the trend between increasing age of injury and each item.

We observed a significant increase in the likelihood of patients experiencing falls on level surfaces, descending stairs, or low-level falls as the age of injury increased. Conversely, a younger age of injury was significantly associated with traffic accidents involving motorcycles, falls from heights, being struck by objects, or sports injuries. Additionally, with increasing age of injury, older patients tended to have injuries at the C1-C4 cervical level, while younger patients exhibited a tendency for more thoracic and lumbar spine injuries.

We have added the above results as a table and modified the Results section.

*Page 14 line 1 in the Results section*

*We observed a significant increase in the likelihood of patients experiencing falls on level surfaces (P < 0.0001), descending stairs (P < 0.0001), or low-level falls (P < 0.0001) as the age of injury increased. Conversely, a younger age of injury was significantly associated with traffic accidents involving motorcycles (P < 0.0001), falls from heights (P < 0.0001), being struck by objects (P = 0.0036), or sports injuries (P < 0.0001). The older the patient was at the time of injury, the more likely they were to consume alcohol (P = 0.0052). Additionally, with increasing age of injury, older patients tended to have injuries at the C1-C4 cervical level (P < 0.0001), while younger patients exhibited a tendency for more thoracic (T1-T6: P = 0.0003; T7-T12: P < 0.0001) and lumbar (L1-L5: P < 0.0001) spine injuries. As the age at the time of injury increased, there was a higher percentage of AIS grade C (P = 0.0132) and D (P = 0.0002) cases, while grade A (P = 0.0006) cases tended to be more common in younger patients (Table 4).*

**3. Gradually getting older, is there a significant difference? It would seem that a statistical difference should be sought.**

We acknowledge the oversight in our statistical analysis and, after consulting with statistical experts, have determined that the chi-square test is not suitable for elucidating trends over time. We agree with the reviewer's suggestion and have opted for regression analysis, which is deemed more appropriate for our study. Specifically, we utilized regression analysis to assess age-related changes and also the Cochran-Armitage test to detect significant differences in the percentage change of each section over time. We express our gratitude to the reviewer for the valuable feedback. The revisions undertaken have substantially enhanced the content of this paper.

*Page 12 line 8 in the Results section*

*We conducted regression analysis to examine changes over time in age at TSCI. The results revealed a statistically significant increasing trend in age at injury (Spearman’s rank correlation coefficient: age at injury vs. calendar year, estimated regression line: Y = 51.741082 + 0.6322536X, P < 0.0001) (Figure 1C).*

*Figure 1C*

**4. The χ2 is mentioned in the analysis method, but it is unclear whether it is actually done or not.**

Thanks to the reviewer's insightful comments, we identified a significant issue in the statistical analysis during the initial submission of our study. In response, we conducted a thorough discussion with a statistical expert group and meticulously reanalyzed the study's results, employing appropriate statistical methods. Specifically, we utilized regression analysis to assess age-related changes and the Cochran-Armitage test to detect significant differences in the percentage change of each section over time. We express our gratitude to the reviewer for the valuable feedback. The revisions undertaken have substantially enhanced the content of this paper.

As shown in Figure 1C, we observed a statistically significant increasing trend in age at injury. For statistical analysis, we opted for regression analysis instead of the chi-square test.

*Figure 1C*

**5. I do not see where there is a significant difference in Table 1, 2, and 3. Please describe the tables more clearly.**

Thank you for emphasizing this crucial point. I recognize the significance of clearly presenting significant differences in this paper. As mentioned earlier, we employed regression analysis to scrutinize such differences, particularly in continuous variables like age. In addition, the Cochran-Armitage test was used for statistical evaluation of changes over time for categorical variables over a 15-year period. Given that Table 1 provides only a patient summary, we deem it unnecessary to examine significant differences there. However, for Tables 2 and 3, p-values have been incorporated to adjust and highlight these differences.

*Table 2*

*Table 3*

**Reviewer #2: This scientific article, titled "Epidemiology and Demographics of Traumatic Spinal Cord Injury in Japan," provides valuable insights into the epidemiology and demographics of traumatic spinal cord injuries (TSCI) in Japan. This article presents a comprehensive epidemiological study of traumatic spinal cord injuries (TSCI) over a 15-year period, focusing on patients treated at the Spinal Injuries Center in Japan. The study reveals a shift in the demographics of TSCI, with a notable increase in elderly patients, highlighting the aging population in Japan. The most frequent spinal level affected was the cervical region, and the severity of injuries ranged from complete to incomplete tetraplegia. Falls emerged as the leading cause of TSCI, with a substantial portion of injuries related to alcohol consumption. The study identifies rising health care challenges and emphasizes the importance of understanding the evolving epidemiology of TSCI for resource management and prevention strategies, especially in the context of aging populations worldwide.
The study provides a comprehensive and detailed analysis of traumatic spinal cord injuries (TSCI) over a 15-year period, making use of data from more than 1,000 patients. The use of a single-institution database ensures consistency and accuracy in data collection and analysis as well as the longitudinal data provides a bigger picture of the issue of TSCI.**

**However, there are some notable flaws in the article that I would like to describe below:**

We appreciate the positive evaluation of our study. By addressing the important points that were raised, the quality of the manuscript was greatly improved. If any concerns of Reviewer 2 remain, we ask for the opportunity to make further revisions.

**Major comments:**

**•There is an evident information bias in the periods where the data was not collected properly. For accurate data analysis you should remove that data from this article. In that same line of ideas, the statistical analysis is not strong enough for the aim and the data use that is described. Using chi2 limits the study comparability and conclusions.**

As pointed out by the reviewer, there was a temporary inability to accept patients in 2011 over the 15-year period, leading to a significant decrease in the number of patients enrolled in the database during that year. However, it is crucial to note that from January 2011 to April 2011, patients were recruited as usual. We believe that excluding patients registered in 2011 is unnecessary, especially since the patient population of accepted patients did not change, and there was no bias in the patient profile during this period. Arbitrarily removing data from 2011 could introduce information bias.

We acknowledge the oversight in our statistical analysis and, after consulting with statistical experts, have determined that the chi-square test is not suitable for elucidating trends over time. We agree with the reviewer's suggestion and have opted for regression analysis, which is deemed more appropriate for our study. Specifically, we utilized regression analysis to assess age-related changes and also the Cochran-Armitage test to detect significant differences in the percentage change of each section over time. We express our gratitude to the reviewer for the valuable feedback. The revisions undertaken have substantially enhanced the content of this paper.

As shown in Figure 1C, we observed a statistically significant increasing trend in age at injury. For statistical analysis, we opted for regression analysis instead of the chi-square test.

*Figure 1C*

**• in the methods section, chi square tests are mentioned. However, there is no p values reported in the results section of this study. Was an analysis carried out?**

As previously mentioned, we made a methodological change from our initial submission, opting for regression analysis and the Cochran-Armitage test. We revised the methods section accordingly, supplementing each statistical result with a p-value.

*Page 11 line 3 in Statistical Analysis section*

*We reported descriptive statistics using proportions for categorical data and means with ranges for continuous data. Additionally, regression analysis was employed for continuous variables such as age, height, weight, and BMI. In contrast, the Cochran-Armitage test was used to analyze trends in qualitative variables, such as proportions. This comprehensive approach enabled us to investigate trends and associations over the 15-year period.*

**• in the results section, is it a percentage of the total patients presenting to the facility? Or is it the total patients with TSCI? Please clarify to avoid confusion. The axis titles can be used to bring this out well.**

In light of the reviewer's comments, we acknowledge that the title of the axis in the initial submission may be misleading to the reader. The percentages listed in this paper reflect the percentages of the total patients with TSCI, not the total patients presenting to the facility. In response to your suggestion, we have revised the titles of the axes in Figures 1 and 2 to make them clearer and to help the reader understand what the percentages mean.

*Figure 1*

*Figure 2*

**• Would it be possible to carry out regression analysis in order to get odds ratios? These may prove to be beneficial to thee analyses and results section. These would also help to account for effect modifiers like operation rates on length of stay.**

In acknowledgment of the reviewer's comment, we understand that the chi-square test does not measure the strength of the association. While logistic regression analysis could capture increased odds ratios due to specific risk factors, our primary focus in this study was to investigate the 15-year trend in patient profiles over time. For this purpose, we believe that linear regression analysis and the Cochran-Armitage test are adequate methods. The results of the revised analysis, in our view, are sufficient to support the conclusions we advocate.

In this study, we employed the chi-square test to assess the association between Neurological Level of Injury (NLI) and alcohol consumption in patients with cervical spinal cord injury, focusing on obtaining odds ratios. When restricting our analysis to patients with cervical cord injuries and NLI at C1-C4 and C5-C8, we observed a significant correlation between alcohol consumption and increased damage in the upper cervical cord (C1-C4) (odds ratio = 1.64, 95% confidence interval 1.13-2.37, p = 0.0084).For other variables, we utilized the Cochran-Armitage trend test to identify significant differences at the p-value level, considering our analysis spanned a 15-year period and aimed at assessing trends.

**• The discussion section could be strenghten up with other studies carried out globally in other aging countries if possible.**

We express our gratitude to the reviewers for their valuable insights. The discussion section has been revised in accordance with the provided comments.

*Page 17 line 15*

*According to reports on the global prevalence and incidence of SCI, the peak age of SCI patients is in the 20s to 40s, with traffic trauma being the most common etiology of injury in Spain, Turkey, and Greece.[6] In a single-center study in China, the age of injury peaked at 40 years, although there was an increasing trend in the age of injury from year to year.[35] The mean age of injury in Scotland was approximately 50 years.[36] There is concern that the age of injury may increase in the future in the above-mentioned low-middle-income countries. In fact, the results of this study show that the distribution of the injured population is shifting to a single peak of 60 to 74 years of age. In Germany, a leading country in terms of aging, the most recent report indicates that the peak age of injury is changing to 75-80 years old.[37] Injury ages and patterns are likely to differ depending on the population distribution and living environment in each country, and the results of this study are expected to reflect the future outlook for other low-middle-income countries.*

**Minor comments:**

**• General
o The language should be adjusted to use "low- and middle-income countries" instead of "developed" or "developing countries" for accuracy, impartiality, and to avoid perpetuating stereotypes and biases associated with the traditional terms.**

Thanks for the important point. In accordance with the reviewer's instructions, we have revised the description as follows

*Page 5 line 14 in the Introduction section*

*Recent published incidences of TSCI have ranged between 12.6 and 86 per million in the high-income countries.*

*Page 16 line 1 in the Discussion section*

*Because populations worldwide are aging, the recent increase in cervical SCI in the elderly will be an important issue that could accelerate the pressure on health care costs in many countries, including currently low- and middle-income countries.*

**• Abstract:
o Data Source Information: The abstract mentions using a locally designed SCI database, but it doesn't describe this database in detail. Readers might be interested in understanding the data collection methods, and its reliability.**

The Japan Single Center Study for Spinal Cord Injury Data Base (JSSCI-DB) used in this study has been previously published in various papers. While we originally planned to reference prior studies for details, in response to the reviewer's comment, we have now incorporated an explanation of the JSSCI-DB in the paper.

*Page 8 line 8 in the Materials and Methods section*

*Data for all patients with TSCIs admitted to the Spinal Injuries Center between May 2005 and December 2021 were collected using a locally designed SCI database known as the Japan Single Center Study for Spinal Cord Injury Database (JSSCI-DB). Initiated in 2005 at the Spinal Injuries Center, the JSSCI-DB serves as a comprehensive repository. An overview of the JSSCI-DB reveals the collection of longitudinal test data for 134 outcome measures, encompassing neurological assessments, physical function assessments, and health-related quality of life assessments.[23, 24]*

**• Introduction:
o Limited Context: While the introduction provides background information about TSCI and its historical trends, it lacks a comprehensive literature review. A thorough review of existing literature would help provide a better context for the study and demonstrate how the current study contributes to the field.**

Through this revision, we have added an additional literature review. In the revised article, we have cited articles on epidemiological studies of spinal cord injury in Japan, the United States, Canada, China, Norway, Italy, Scotland, and Germany, along with references to global epidemiological evaluations. Based on the reviewers' advice, we have made additional revisions to the introduction.

*Page 6 line 8*

*Reports on the worldwide prevalence and incidence of SCIs indicate significant variations in annual incidence rates, sex ratios, patient age distributions, injury mechanisms, and neurological levels of injury across different countries. Past literature has highlighted Japan as having a notably higher age range among SCI patients compared to other nations.[6] The age-standardized incidence rate of SCI varies based on the socio-demographic Index, reflecting the socioeconomic development level of each country.[7] Developing regions such as South Asia, Saharan Africa, Latin America, the Caribbean, North Africa, and the Middle East experience dramatic linear population growth. In contrast, countries like Japan, with low fertility and mortality rates, exhibit a distinct demographic composition. Consequently, the epidemiology of SCI may differ between low-income and high-income countries. It is highly likely that fertility and mortality rates will decrease in the future in such low-income countries. Therefore, epidemiological studies, especially those in Japan, with unique demographic characteristics, become crucial for understanding global health trends and shaping future healthcare strategies.*

**o The introduction goes from epidemiology to Japan and back to epidemiological data. Restructuring the introduction to commence with the definition, followed by an exploration of epidemiology and its associated costs, then delving into the relevance of the study within the local context of Japan (single center), addressing the knowledge gap, and finally elucidating the study's objectives and its broader significance, may enhance the overall introduction.**

Thank you for your invaluable advice. Considering the reviewer's comments, I have reorganized the introduction section in the following order: commencing with the definition of SCI, followed by an exploration of epidemiology and its associated costs, then delving into the relevance of the study within the local context of Japan, addressing the knowledge gap, and finally, elucidating the study's objectives and its broader significance. We hope you will review the revised manuscript. If additional revisions to the introduction are required, we will be happy to make the necessary adjustments to the manuscript.

**o The introduction can be edited to include a small section on some of the debilitating complications associated with TSCI in order to show the great effect such injuries have on the QOL of patients**

In response to the reviewer's comments, a description of the significant impact of spinal cord injury on the patient's quality of life has been added to the introduction.

*Page 5 line 20*

*Permanent motor, sensory, and autonomic impairments that occur after TSCI have a significant negative impact on the lifestyle of patients and their families. Urinary tract infections, pneumonia, pressure ulcers, and depression resulting from neurological insult can lead to the deterioration of physical and mental health. The more severe the neurological impairment, the longer the rehabilitation required for the SCI patients to return to daily life.*

**• Materials and Methods:
o In order to enhance the methodology section and ensure comprehensive and transparent reporting, incorporating the STROBE guidelines to restructure the content is advisable.** [**https://www.strobe-statement.org/checklists/**](https://www.strobe-statement.org/checklists/)

We appreciate the reviewer's guidance and have revised the methodology section to align with the STROBE guidelines.

*Page 10 line 14 in the Materials and Methods section*

*Age, height, weight, and BMI were considered as continuous variables, while age group, sex, mechanism of injury, AIS, NLI, operation rate, alcohol consumption at injury, and category of SCI (tetraplegia and paraplegia) were presented as percentage and treated as categorical variables.*

*Page 11 line 3 in the Statistical Analysis section*

*We reported descriptive statistics using proportions for categorical data and means with ranges for continuous data. Additionally, regression analysis was employed for continuous variables such as age, height, weight, and BMI. In contrast, the Cochran-Armitage test was used to analyze trends in qualitative variables, such as proportions. This comprehensive approach enabled us to investigate trends and associations over the 15-year period. We employed a chi-square test to assess the association between alcohol consumption and the NLI in patients with cervical spinal cord injury.*

*STROBE Statement*

**o Incomplete Data Description: The section provides details about data collection but lacks information about sample size justification, data quality control measures, and ethical considerations. These omissions affect the transparency and replicability of the study.**

As outlined in the introduction section, the Japan Single Center Study for Spinal Cord Injury Database (JSSCI-DB) commenced patient enrollment in 2005. Clinical data for all spinal cord injury patients transported to our hospital are recorded, with the exception of cases where patient consent was not obtained. Given that we exclusively gather information pertaining to spinal cord injury patients, we deem it unnecessary to provide a justification for the sample size.

As the reviewer emphasized, maintaining data quality is crucial for accuracy and consistency across the database. Various factors, including correct formatting and identification of invalid values, are considered in the assessment of data quality. In the JSSCI-DB, dedicated personnel conduct regular data quality control. The patients' profiles and neurological data used in this study were confirmed after database registration. Determining the American Spinal Injury Association (ASIA) Impairment Scale (AIS) and the spinal or neurological level of injury (NLI) is particularly challenging and requires assessment by trained personnel. As indicated in the initial submission, neurological findings and final diagnoses were established by physicians and physician assistants who completed the ASIA International Standards Training e-Learning Program (InSTeP), as well as in-person training.

*Page 8 line 14 in the Materials and Methods section*

*Various factors, including correct formatting and identification of invalid values, are considered in the assessment of data quality. In the JSSCI-DB, dedicated personnel conduct regular data quality control. The patients' profiles and neurological data used in this study were confirmed after database registration.*

We have also included a description of ethical considerations in the methods section.

*Page 8 line 17 in the Materials and Methods section*

*This study was approved by the Ethical Review Board of Japan Labor Health and Welfare Organization Spinal Injuries Center (Approval code: 16-7). We had all the necessary consent from the patients involved in the study, including consent to participate in the study.*

**o It says: “May 2011 to December 2011, patient data could not be entered into the database because of hospital renovations; therefore, the number of registered patients decreased considerably in 2005 and 2011” but it is not clear how a renovation in May 2011 would affect the data in 2015. This is maybe a typo.**

Upon reviewing the comments provided by the reviewer, I recognized that the initial submission of my manuscript could potentially mislead readers. Consequently, we have revised the description as follows.

*Page 9 line 23 in the Materials and Methods section*

*Between May 2011 and December 2011, the entry of patient data into the database was hindered by hospital renovations. As a result, the number of registered patients considerably decreased in 2011.*

**o Bias: The section should include a discussion of potential limitations of the study. For instance, the lack of complete data for certain years and the exclusion of pediatric cases are mentioned in the conclusion but should be included here for clarity.**

As pointed out by the reviewer, we addressed the possibility of bias in subject selection for the study in the methods section.

*Page 10 line 1*

*This study may exhibit variations in the characteristics of the target population due to a temporary suspension of patient admissions in 2011 and a low number of pediatric spinal cord injury case admissions, potentially resulting in selection bias.*

**o Statistical Analysis: trends should not be analyzed with chi-squared test. More elaborated analysis including regressions (going from linear regressions to time-series analysis) would help to really understand the differences in the periods and the rates of occurrence of the outcome variables, which should be more clearly specified.**

Thank you for your valuable feedback. In light of the reviewer's comments, we acknowledge that the chi-square test was inappropriate for assessing trends. Consequently, we utilized regression analysis for examining trends in continuous variables and the Cochran-Armitage test for categorical variables. Revisiting the statistical analysis based on the reviewers' input has provided us with confidence that our evaluation is now more appropriate. We believe these adjustments enhance the clarity and understanding of the paper for readers.

We have changed the description of the section on statistical analysis as follows.

*Page 11 line 3 in the Statistical Analysis section*

*We reported descriptive statistics using proportions for categorical data and means with ranges for continuous data. Additionally, regression analysis was employed for continuous variables such as age, height, weight, and BMI. In contrast, the Cochran-Armitage test was used to analyze trends in qualitative variables, such as proportions. This comprehensive approach enabled us to investigate trends and associations over the 15-year period.*

**o The chi square test ideally indicates significance of association and not the strength of association as mentioned in the statistical analysis section in the methods.**

Thank you for your valuable feedback. We acknowledge the correctness of the reviewer's comments. Indeed, the chi-square test does not indicate the strength of association. In response, we have chosen not to mention the strength of the association in this paper, as our focus is mainly on assessing trends over time.

**• Results:
o Considering the feedback provided in the methods section, it is evident that additional effort is needed, particularly in the statistical analysis portion of the results.**

Thank you for highlighting this crucial point. We acknowledge that we initially fell short in the statistical analysis of the paper. In response, we invited a statistician to join us as a co-author, and together, we engaged in extensive discussions to refine the analysis. We made significant efforts to address the statistical analysis by incorporating regression analysis and the Cochran-Armitage trend test, striving to enhance the paper.

**o Please harmonize the legends of Figure 1. There seems to be an error whereby E is captioned twice.**

We apologize for the careless oversight. Thanks to the reviewer, we have corrected the text you indicated as follows.

*Page 29 line 12*

*(D) Percentages of TSCI by age group from 2005 to 2021. The 60-74 age group was the most common group, and the 75+ age group was the second most common, beginning in 2010.*

*(E) Percentages of TSCI by neurological level of injury (NLI) from 2005 to 2021. The most frequent NLI affected by the injury was the high cervical level (C1-C4), followed by the low cervical level (C5-C8).*

**• Discussion:
o Incomplete Explanation of Findings: The discussion section provides some interpretation of the data but lacks a comprehensive analysis of the trends observed in the study. A more detailed discussion of how the changing demographics of TSCI patients might impact healthcare resources and strategies is needed.**

In response to the reviewer's valuable feedback, we have incorporated additional discussion points into our work.

*Page 18 line 3*

*According to the results of this study, there is a noteworthy increasing trend in the age at injury among TSCI patients over time. Additionally, there is a significant rise in the incidence of certain mechanisms of injury, particularly falls on level ground, falls downstairs, and falls from low heights. Conversely, there is a decline in sports-related injuries among young individuals, which previously constituted a substantial proportion of SCI cases. With the ongoing global progression toward an aging society, it is highly anticipated that the number of SCI patients in the elderly demographic will rise significantly.[7] Consequently, it is imperative to develop future treatment strategies for elderly SCI patients, taking into account aspects such as surgical interventions, rehabilitation, and preventive medicine.*

**o Minor grammatical mistakes in conclusion as highlighted.**

The text was revised according to the reviewer's instruction.

*Page 20 line 6 in the Conclusion section*

*Since 2014, the average age at injury has gradually increased to exceed 60 years.*

**o Please add the fact that this is a single center study and that limits generalizability. A similar issue was mentioned in the introduction when arguing that the incidence of TSCI varies from region to region even within the same country.**

In agreement with the reviewer's comments, we acknowledge certain limitations to the generalizability of the data presented in this study. To address these concerns, a specific description has been included in the Discussion section to provide a more comprehensive understanding of the scope and applicability of our findings.

*Page 18 line 19 in the Discussion section*

*This study is a single-center analysis and hence may have limited generalizability.*

**o Limited Discussion of Alcohol Use: Is this study really addressing this issue? While the study mentions the impact of alcohol on TSCI, there is a lack of discussion about potential interventions or policy recommendations related to alcohol use as a preventive measure.**

Thank you for the important remarks. As indicated in Table 3 of the initial submission, 13.2% to 19% of patients were reported to have consumed alcohol at the time of spinal cord injury in our 15-year epidemiological study.

We also analyzed the percentage of alcohol consumption within each age group. While the Cochran-Armitage trend test doesn't provide odds ratios, our findings revealed a significant increase in the percentage of alcohol consumption among older age groups.

We further explored the association between neurological level of injury (NLI) and alcohol consumption. When restricting our analysis to patients with cervical cord injuries and NLI at C1-C4 and C5-C8, we observed a significant correlation between alcohol consumption and increased damage in the upper cervical cord (C1-C4) (odds ratio = 1.64, 95% confidence interval 1.13-2.37, p = 0.0084).

Older patients at the time of injury tend to consume more alcohol, and those who consume alcohol are at a higher risk of increased injury in the upper cervical spinal cord. This suggests that reducing excessive alcohol consumption may mitigate the risk of developing severe spinal cord injury. We have incorporated these findings into the paper, and the discussion has been revised accordingly.

*Page 15 line 21 in the Discussion section*

*Patients with cervical SCI who consumed alcohol at the time of injury had a 1.64 times greater risk of upper cervical SCI with NLI of C1-C4. Therefore, avoiding excessive alcohol consumption may help prevent SCIs in the elderly. Additionally, reducing the risk of upper cervical SCI could potentially lead to a reduction in healthcare costs.*

**o Future Research: The discussion section should conclude with suggestions for future research, building on the findings and limitations of the current study.**

In response to the reviewer's comments, we have carefully revised the concluding section of the discussion, incorporating specific adjustments and enhancements to address the raised concerns and provide a more robust and insightful conclusion.

*Page 18 line 23 in the Discussion section*

*The age at SCI is anticipated to rise globally in the future, emphasizing the need to establish preventive measures and treatment strategies for SCIs in the elderly. Additionally, exploring the potential impact of interventions aimed at controlling excessive alcohol consumption and preventing falls on reducing the incidence of SCI is desirable.*

**• Conclusion:
o Incomplete Summary of Findings: The conclusion should provide a concise summary of the key findings and their implications for the field of TSCI research and healthcare policy. This would help readers understand the study's significance more clearly.**

Thank you for your meaningful comments. We have revised the Conclusion section as per the reviewer's instructions and welcome any additional feedback you may have.

*Page 20 line 2 in the Conclusion section*

*To accurately determine the epidemiology of SCI patients, it is necessary to establish an environment that allows for the long-term, consistent follow-up of patients in specialized facilities by professional staff. This study presents the prospective findings of the demographics and epidemiology of TSCI in single center in Japan over a period of more than 15 years since 2005. Throughout the study period, approximately 80% of the patients were male.* *Since 2014, the average age at injury has gradually increased to exceed 60 years. As the number of injuries among the elderly increased, the injury mechanisms shifted from high-fall trauma and traffic accidents to falls on level surfaces and downstairs. Incomplete tetraplegia in the elderly due to upper cervical SCI also increased over time. Avoiding excessive alcohol consumption may help prevent SCIs in the elderly. Given that Japan has one of the highest aging populations in the world, epidemiological studies in this country will be very helpful in determining health insurance and medical costs and deciding treatment strategies for SCI in future aging populations worldwide.*

**o Please complete your conclusion. Line 4 has an incomplete sentence.**

We apologize for the careless oversight. Thanks to the reviewer, we have corrected the text you indicated as follows.

*Page 20 line 6 in the Conclusion section*

*Throughout the study period, approximately 80% of the patients were male.*

**In summary, this scientific article provides valuable data on the epidemiology and demographics of TSCI in Japan, but it contains several flaws related to clarity, completeness, and context. Addressing these issues would enhance the quality and impact of the research.**

I would like to express my heartfelt thanks to the reviewers for their invaluable remarks. Thanks to their essential comments, I believe that the revised paper has significantly improved, enhancing the reader's understanding of its significance. Should further revisions be necessary, I am more than willing to make the necessary adjustments.

**Academic Editor’s Comments to Author**

**1. Page 3 line 21 in the Abstract section**

**most common cause or etiology**

In accordance with the reviewer's instructions, the text has been modified as follows.

*Page 4 line 14 in the Abstract section*

*Incomplete tetraplegia was the most common cause or etiology category of TSCI, accounting for 48.4% of cases.*

**2. Page 5 line 1 in the Introduction section**

**Please include a small section on complications of SCI and how this negatively impacts QOL among patients to show the importance of carrying out this study**

Following the reviewer's comments, we added a description to the introduction.

*Page 5 line 20 in the Introduction section*

*Permanent motor, sensory, and autonomic impairments that occur after TSCI have a significant negative impact on the lifestyle of patients and their families. Urinary tract infections, pneumonia, pressure ulcers, and depression resulting from neurological insult can lead to the deterioration of physical and mental health. The more severe the neurological impairment, the longer the rehabilitation required for the TSCI patient to return to daily life.*

**3. Page 7 line 11 in the Materials and Methods section**

**This repetition can be avoided by combining the 2 sentences**

In accordance with the reviewer's instructions, the text has been modified as follows.

*Page 8 line 21 in the Materials and Methods section*

*We retrospectively reviewed the dataset for research purposes from July 1 to October 31, 2022.*

**4. Page 7 line 13 in the Materials and Methods section**

**Is this within 4 weeks from injury or fall or 4 weeks from manifestation of disease?**

Additional information has been incorporated in response to the reviewer's advice.

*Page 8 line 22 in the Materials and Methods section*

*Only patients admitted to our institution within four weeks from injury or fall were included.*

**5. Page 7 line 20 in the Materials and Methods section**

**Were patients with comorbidities excluded as some diseases may affect length of stay?**

As stated in the initial submission, patients with complications such as tumors, infections, vascular abnormalities, cerebrovascular disease, and psychogenic paralysis, who exhibited neurological disorders making it difficult to evaluate the pathology of spinal cord injury, were excluded from the study. Patients with comorbidities that did not directly affect muscle weakness or sensory impairment were included in this study.

Following the reviewer's advice, I revised the description to provide a more detailed explanation of the excluded and eligible patients.

*Page 9 line 4 in the Materials and Methods section*

*Patients with neurological deficits secondary to nontraumatic conditions, such as tumors, infections, vascular abnormalities, cerebrovascular disease, and psychogenic paralysis, who exhibited neurological disorders making it difficult to evaluate the pathology of SCI, were excluded from the study. Patients with medical comorbidities, such as hypertension, diabetes, or heart disease, that did not directly affect severe neurological disorders, were included in this study.*

**6. Page 8 line 16 in the Materials and Methods section**

**Please define how you determined complete vs incomplete paraplegia and tetraplegia to allow for reproducibility of the study**

Complete tetraplegia indicates patients with cervical spinal cord injuries classified as AIS A, displaying paralysis in both upper limbs and complete paralysis in both lower limbs. Cases of complete paraplegia describe patients with no upper limb paralysis but complete paralysis in both lower limbs. Incomplete tetraplegia includes patients with cervical spinal cord injuries encompassing AIS B, C, or D; these individuals exhibit incomplete paralysis in both upper and lower limbs. Incomplete paraplegia refers to patients with no upper limb paralysis but partial paralysis in both lower limbs. Previous literature on the epidemiology of spinal cord injuries has also categorized cases into complete vs. incomplete paraplegia and tetraplegia (Halvorsen et al., 2019; Lenehan et al., 2012; Noonan et al., 2012). In response to reviewer comments, we recognized insufficient explanation in the initial manuscript and have thus modified the Introduction section and the Material and Method section accordingly.

*Page 5 line 4 in the Introduction section*

*Paraplegia is associated with damage to the thoracic, lumbar, or sacral spinal cord, while tetraplegia occurs with damage to the cervical cord.*

*Page 10 line 7 in the Materials and Methods section*

*Complete tetraplegia indicates patients with cervical SCIs classified as AIS A, displaying paralysis in both upper limbs and complete paralysis in both lower limbs. Cases of complete paraplegia describe patients with no upper limb paralysis but complete paralysis in both lower limbs. Incomplete tetraplegia includes patients with cervical SCIs encompassing AIS B, C, or D; these individuals exhibit incomplete paralysis in both upper and lower limbs. Incomplete paraplegia refers to patients with no upper limb paralysis but partial paralysis in both lower limbs.*

**7. Page 9 line 1 in the Statistical Analysis section**

**Would it be possible to carry out regression analysis in order to obtain odds ratios that would further beef up the results section of this study**

In acknowledgment of the reviewer's comment, we understand that the chi-square test does not measure the strength of the association. While logistic regression analysis could capture increased odds ratios due to specific risk factors, our primary focus in this study was to investigate the 15-year trend in patient profiles over time. For this purpose, we believe that linear regression analysis and the Cochran-Armitage test are adequate methods. The results of the revised analysis, in our view, are sufficient to support the conclusions we advocate.

In this study, we employed the chi-square test to assess the association between Neurological Level of Injury (NLI) and alcohol consumption in patients with cervical spinal cord injury, focusing on obtaining odds ratios. When restricting our analysis to patients with cervical cord injuries and NLI at C1-C4 and C5-C8, we observed a significant correlation between alcohol consumption and increased damage in the upper cervical cord (C1-C4) (odds ratio = 1.64, 95% confidence interval 1.13-2.37, p = 0.0084).For other variables, we utilized the Cochran-Armitage trend test to identify significant differences at the p-value level, considering our analysis spanned a 15-year period and aimed at assessing trends.

**8. Page 9 line 3 in the Statistical Analysis section**

**Some descriptive statistics were also presented as means and ranges**

The description has been modified in response to the reviewer's feedback.

*Page 11 line 3 in the Statistical Analysis section*

*We reported descriptive statistics using proportions for categorical data and means with ranges for continuous data.*

**9. Page 9 line 5 in the Statistical Analysis section**

**Chi squares by themselves do not test for strength of association but rather just show associations between variables**

**It would be beneficial to include regression analyses in order to capture increased odds ratios due to certain risk factors**

In acknowledgment of the reviewer's comment, we understand that the chi-square test does not measure the strength of the association. While logistic regression analysis could capture increased odds ratios due to specific risk factors, our primary focus in this study was to investigate the 15-year trend in patient profiles over time. For this purpose, we believe that linear regression analysis and the Cochran-Armitage test are adequate methods. The results of the revised analysis, in our view, are sufficient to support the conclusions we advocate.

**10. Page 12 line 1 in the Discussion section**

**Is it possible to discuss findings in light of other aging populations such as Germany if possible?**

We have revised the discussion as directed by the reviewer, incorporating insights from the most recent article on the epidemiology of spinal cord injury in Germany (Rau et al., European Spine Journal, 2023).

*Page 17 line 22 in the Discussion section*

*In Germany, a leading country in terms of aging, the most recent report indicates that the peak age of injury is changing to 75-80 years old.[37] Injury ages and patterns are likely to differ depending on the population distribution and living environment in each country, and the results of this study are expected to reflect the future outlook for other low-middle-income countries.*

**11. Page 12 line 22 in the Discussion section**

**Having odds ratios would have further strengthened this point**

Thank you for the important point. As indicated in Table 3 of the initial submission, 13.2% to 19% of patients were reported to have consumed alcohol at the time of spinal cord injury in our 15-year epidemiological study.

We also analyzed the percentage of alcohol consumption within each age group. While the Cochran-Armitage trend test doesn't provide odds ratios, our findings revealed a significant increase in the percentage of alcohol consumption among older age groups.

We further explored the association between neurological level of injury (NLI) and alcohol consumption. When restricting our analysis to patients with cervical cord injuries and NLI at C1-C4 and C5-C8, we observed a significant correlation between alcohol consumption and increased damage in the upper cervical cord (C1-C4) (odds ratio = 1.64, 95% confidence interval 1.13-2.37, p = 0.0084).

Older patients at the time of injury tend to consume more alcohol, and those who consume alcohol are at a higher risk of increased injury in the upper cervical spinal cord. This suggests that reducing excessive alcohol consumption may mitigate the risk of developing severe spinal cord injury. We have incorporated these findings into the paper, and the discussion has been revised accordingly.

*Page 14 line 12 in the Results section*

*We further explored the association between neurological level of injury (NLI) and alcohol consumption. When restricting our analysis to patients with cervical cord injuries and NLI at C1-C4 and C5-C8, we observed a significant correlation between alcohol consumption and increased damage in the upper cervical cord (odds ratio = 1.64, 95% confidence interval 1.13-2.37, p = 0.0084) (Supplemental Table 1).*

*Page 15 line 21 in the Discussion section*

*Patients with cervical SCI who consumed alcohol at the time of injury had a 1.64 times greater risk of upper cervical SCI with NLI of C1-C4. Therefore, avoiding excessive alcohol consumption may help prevent SCIs in the elderly. Additionally, reducing the risk of upper cervical SCI could potentially lead to a reduction in healthcare costs.*

**12. Page 14 line 12 in the Discussion section**

**Please add the limitation that this is a single center study and hence may have limited generalizability**

In accordance with the reviewer's instructions, we have incorporated the following text into the discussion section.

*Page 18 line 19 in the Discussion section*

*This study is a single-center analysis and hence may have limited generalizability.*

**13. Page 14 line 21 in the Discussion section**

**Please edit this sentence for flow and grammar**

In response to the reviewer's instructions, we have revised our description.

*Page 18 line 21 in the Discussion section*

*Despite these limitations, this study represents large single-center data from Japan, a representative of an aging society, and will help develop future treatment strategies for SCI patients in an aging society worldwide.*

**14. Page 15 line 5 in the Conclusion section**

**Incomplete sentence**

We apologize for the careless oversight. Thanks to the reviewer, we have corrected the text you indicated as follows.

*Page 20 line 6 in the Conclusion section*

*Throughout the study period, approximately 80% of the patients were male.*

**15. Page 15 line 5 in the Conclusion section**

**gradually increased to**

The text was revised according to the reviewer's instructions.

*Page 20 line 6 in the Conclusion section*

*Since 2014, the average age at injury has gradually increased to exceed 60 years.*

**16. Page 15 line 9 in the Conclusion section**

**Were statistical tests run to conclude this? Please present the p values**

In this round of revision, we identified an association between age at injury and alcohol consumption in patients with SCI, uncovering a tendency for alcohol consumption among patients with cervical SCI, which are more prevalent in the elderly. Although not directly proving this statement, we believe the research results presented in this paper are sufficient to support recommendations for future SCI prevention measures.

**17. Page 24 line 11 in the Figure Legends section**

**E is captioned twice. Please resolve.**

We apologize for the careless oversight. Thanks to the reviewer, we have corrected the text you indicated as follows.

*Page 29 line 12*

*(D) Percentages of TSCI by age group from 2005 to 2021. The 60-74 age group was the most common group, and the 75+ age group was the second most common, beginning in 2010.*

*(E) Percentages of TSCI by neurological level of injury (NLI) from 2005 to 2021. The most frequent NLI affected by the injury was the high cervical level (C1-C4), followed by the low cervical level (C5-C8).*

**18. Figure 1B in Page 29**

**Is this the total patients presenting to the facility or is this the patients with SCI. Please clarify. It can be misleading to the reader.**

Thanks to the reviewer's suggestion, we have realized that the figures in the initial submission could be misleading to readers. As a result, we have revised the figure to make it clearer to readers what they represent.

*Figure 1B*
