# Supplementary material for: Changing trends in traumatic spinal cord injury in an aging society: Epidemiology of 1152 cases over 15 years from a single center in Japan
Source: PLoS One. 2024 May 16;19(5):e0298836. doi: 10.1371/journal.pone.0298836 (PMC11098516; doi:10.1371/journal.pone.0298836)
Supplement: Attachment — Submitted filename: Response to Reviewers.docx [file pone.0298836.s009.docx]

To the editor and reviewers:

We appreciate the reviewer’s constructive comments that significantly improved the quality of the manuscript. We have addressed each reviewer’s comments point-by-point and would appreciate the opportunity to further clarify any questions that remain after this round of revisions. The reviewers’ comments are shown in **bold face** and are followed by our responses. The changes made in the revised manuscript are indicated in underlined italic text.

**Additional Editor Comments:**

**Both reviewers see the contribution from this study to the literature. They recommend to further the discussion by comparing the data from this study with similar studies from other countries. The editor agrees that these comparisons would increase the impact of this study.**

Thank you for your important remarks. As the editor and reviewer indicated, we have further developed our discussion by comparing the results of this study with those of similar studies in other countries. We believe that these changes have significantly enhanced the impact of this paper. We also strongly hope that through these revisions, this paper will meet the criteria for publication in *PLOS ONE*.

**Reviewer(s)' Comments to Author:**

**Reviewer #1: (No Response)**

**Reviewer #2: Dear Author,
Thank you for the revisions made to your manuscript, "Changing trends in traumatic spinal cord injury in an aging society: epidemiology of 1152 cases over 15 years from a single center in Japan." The improvements are noticeable and commendable.**

We thank Reviewer 2 for the critical evaluation of the manuscript. By addressing the reasonable and insightful comments from the reviewer, the manuscript has been significantly improved.

**However, to enhance the publishability of your article, I recommend expanding its scope beyond Japan, a high-income country, to include perspectives from low- and middle-income countries (LMICs). This would enrich the global epidemiological discussion and make your findings more relevant internationally. Incorporating studies like the one found at PubMed (**[**https://pubmed.ncbi.nlm.nih.gov/34035224/**](https://pubmed.ncbi.nlm.nih.gov/34035224/)**), which discusses similar issues in South America and other LMICs, could provide valuable insights into how different socioeconomic contexts affect traumatic spinal cord injuries. This addition would significantly broaden the appeal and applicability of your study.**

Thank you for your important comments. As the reviewer mentions, our results and previous reports have shown that the epidemiology of TSCI differs greatly between high-income countries such as Japan and low- and middle-income countries. In particular, the epidemiological study of TSCI in Colombia (Giraldo et al., *Spinal Cord Series and Cases*, 2021), representative of the low-income countries the reviewer presented, showed that the frequency of SCI caused by interpersonal violence is extremely high in such regions. It is noteworthy that gun violence is the most common etiology among interpersonal violence. Considering these results, we believe that the prevention of SCI should be tailored to the specific mechanisms prevalent in each country.

In response to the reviewers' comments, we have decided to incorporate a discussion on this aspect.

*Page 18 line 12 in the Discussion section*

*A global survey of TSCI has highlighted significant differences in the mechanisms of injury between high-income and low- to middle-income countries.[6] Our findings and previous reports showed that the majority of TSCI result from falls on level ground in Japan,[11, 12] while low- to middle-income countries experience a particularly high frequency of TSCI caused by violence. An epidemiological study in Colombia revealed that interpersonal violence is the most common mechanism of TSCI in the region.[38] Notably, the severity of TSCI resulting from interpersonal violence often leads to a high percentage of ASIA grade A (complete paralysis). In low- to middle-income countries, poor outcomes are exacerbated by factors such as prolonged transport times for injured patients, limited access to intensive care units for treatment, and a high proportion of treatment costs.[39, 40] Strikingly, 90% of these injuries in Colombia are attributed to gun violence. In response, Colombia is implementing a municipal gun control program with the hope of developing more effective methods in the future. In Japan, successful prevention strategies involve awareness-raising activities for fall prevention and improvements in the living environment. However, in low- to middle-income countries with diverse causes of TSCI, the development of entirely different prevention strategies is imperative.*
